# Methods for the Cost-Effective Production of Bacteria-Derived Double-Stranded RNA for *in vitro* Knockdown Studies

**DOI:** 10.3389/fphys.2022.836106

**Published:** 2022-04-13

**Authors:** Thomas-Wolf Verdonckt, Jozef Vanden Broeck

**Affiliations:** Molecular Developmental Physiology and Signal Transduction Research Group, Animal Physiology and Neurobiology Division, Department of Biology, KU Leuven, Leuven, Belgium

**Keywords:** DsRNA (Double-stranded RNA), HT115 strain, insect, lepidoptera, RNAi–RNA interference, pest control

## Abstract

RNA interference (RNAi) is a highly conserved pathway for the post-transcriptional regulation of gene expression. It has become a crucial tool in life science research, with promising potential for pest-management applications. To induce an RNAi response, long double-stranded RNA (dsRNA) sequences specific to the target gene must be delivered to the cells. This dsRNA substrate is then processed to small RNA (sRNA) fragments that direct the silencing response. A major obstacle to applying this technique is the need to produce sufficiently large amounts of dsRNA in a very cost-effective manner. To overcome this issue, much attention has been given to the development and optimization of biological production systems. One such system is the *E. coli* HT115 strain transformed with the L4440 vector. While its effectiveness at inducing knockdowns in animals through feeding of the bacteria has been demonstrated, there is only limited knowledge on the applicability of bacteria-derived dsRNA for *in vitro* experiments. In this paper, we describe and compare methods for the economical (43.2 €/mg) and large-scale (mg range) production of high-quality dsRNA from the HT115 bacterial system. We transformed the bacteria with constructs targeting the *Helicoverpa*-specific gene *Dicer2* and, as a non-endogenous control, the *Green Fluorescent Protein* gene (*GFP*). First, we compared the total RNA extraction yields of four cell-lysis treatments: heating, lysozyme digestion, sonication, and a control protocol. Second, we assessed the quality and purity of these extracted dsRNAs. Third, we compared methods for the further purification of dsRNAs from crude RNA extracts. Finally, we demonstrated the efficiency of the produced dsRNAs at inducing knockdowns in a lepidopteran cell line. The insights and results from this paper will empower researchers to conduct otherwise prohibitively expensive knockdown studies, and greatly reduce the production times of routinely or large-scale utilized dsRNA substrates.

## 1 Introduction

RNA interference (RNAi) is a highly conserved pathway that has become an important tool for loss of function research in both clinical and fundamental research. It enacts post-transcriptional gene silencing through the sequence-specific sequestration or degradation of mRNAs. This process is mediated through an RNA-induced silencing complex (RISC) with the main effector protein being a member of the argonaute family. The RISC is directed to the target mRNA by a complementary small-RNA (sRNA). In arthropods, the cytoplasmic presence of dsRNA activates the small-interfering RNA (siRNA) mediated RNAi (siRNAi) pathway. This siRNAi pathway thus allows the targeted knockdown of genes through the delivery of sequence-specific dsRNA to animal or cellular model systems (Zotti and Smagghe 2015).

The RNAi technology is being developed as a promising biofriendly alternative to current insect pest management strategies (Mezzetti et al., 2020). To this end, the most direct in-field application procedure consists in the spraying of “naked”, complexed, or encapsulated dsRNA onto the host plants or pest insects. It is estimated that such treatments would require 2–10 g of dsRNA per hectare (Zotti et al., 2018).

The efficacy of RNAi is highly dependent on the targeted insect species, tissues and genes. The order of Lepidoptera, comprising moths and butterflies, includes many of the most damaging pest species to agriculture. This insect order is characterized by its refractoriness to the application of RNAi technology (Terenius et al., 2011), partly due to the presence of RNAi efficiency-related nucleases (REases) that degrade dsRNA substrates before they can be processed by the RNAi machinery (Singh et al., 2017; Guan et al., 2018). For these species, several micrograms of dsRNA are required to achieve significant knockdowns when delivered to the hemocoel under experimental settings, with in-field applications expected to be even more demanding (Xu et al., 2016).

A crucial requirement for the application of the RNAi technology in the lab or field is therefore the cost-effective production of dsRNA. Three main approaches are commonly used to produce dsRNA in experimental settings. Synthesis from NTPs is a rapidly advancing technology, with industrial costs as low as ∼$60 USD per Gram (Zotti et al., 2018). A second approach is *in vitro* synthesis through RNA-dependent RNA polymerases. The MEGAscript™ RNAi Kit (Thermo Scientific) allows to produce dsRNA at a cost of ∼$3000 USD for 10 mg. A third option is the production of dsRNA through fermentation. In this process, the dsRNA is synthetized in transgenic cells. This is expected to become the most available and cost-effective method for the large-scale production of dsRNA in the laboratory setting, with target costs near $4 USD per 1 g (Zotti et al., 2018). Still, these prices are yet to become commercially feasible, and custom orders for *in vitro* transcription (IVT) constructs up to 500 bp in size are still billed at ≥$500 USD for 10 mg.

One such system is the bacterial HT115 (DE3) (Takiff et al., 1989) strain of *E. coli*, an RNase III-deficient bacterium. As such, it cannot degrade dsRNA, which allows for its accumulation in the cytoplasm. It can express the bacteriophage T7 polymerase gene from an inducible (Lac) promoter. When transformed with expression plasmids containing T7 promoter sequences, HT115 can produce large quantities of sequence-specific dsRNAs. The L4440 plasmid (Timmons and Fire, 1998) contains two T7 promoters flanking a multiple cloning site and an ampicillin resistance gene.

HT115 bacteria were first transformed with the L4440 plasmid in 2003 (Tenllado et al., 2003) and have since then become a popular tool for functional studies in invertebrate physiology. To this end, the kinetics of dsRNA production in batch cultures have been assayed and production optimized (Papić et al., 2018).

The system is mainly used for direct feeding of induced bacteria to research animals in a process termed bacterium-mediated RNA interference (bmRNAi) (Timmons and Fire, 1998; Goodfellow et al., 2019; Kunte et al., 2020). Recent publications reaffirm its useability in the important lepidopteran pest genera *Spodoptera* and *Helicoverpa* (Vatanparast et al., 2021; Wan et al., 2021; Wang et al., 2021).

For functional research, it is very desirable to produce cost-effective dsRNAs that can be administered via injection or transfection to the research organism. Delivery of live or inactivated bacteria into the hemocoel of orthopteran insects was shown to trigger a knockdown of the target gene (Vogel, 2020). This approach is however unpractical in *Helicoverpa* pests due to the immune responses generated upon the presence of bacteria in the hemolymph (Li et al., 2019). Injections of HT115 bacteria-derived dsRNA extracted using commercial kits was shown to be effective at inducing target gene knockdowns in a lepidopteran species (Wan et al., 2021). As a result, research has focused on developing a cost-effective protocol for the isolation of dsRNA from the bacterial cells. A one-step protocol was published for the extraction of total nucleic acids from the bacteria using cost-effective reagents (Posiri et al., 2013). Still, the one-step phenol-guanidine based protocol (Chomczynski and Sacchi, 1987) is often favored for its RNA-isolation specificity.

Increased physiological effects observed during *in vivo* studies suggest that heating or sonication of bacteria facilitates release of dsRNA into the gut lumen, likely by compromising the integrity of the bacterial cell wall or fragmenting the dsRNA constructs (Kim et al., 2015; Vatanparast and Kim, 2017). Ahn et al. demonstrated that these pre-treatments likewise increase the total RNA yield during phenol-guanidine extractions (Ahn et al., 2019). However, to date, little is known about the reliability of these and other pre-treatments, with no replicate data available, nor is it known how these treatments impact the efficacy of extracted dsRNA at inducing an RNAi response.

The total RNA extracted from the bacterial cells needs to be purified for application in knockdown experiments. Two main techniques are described in literature. The first is digestion of non-specific nucleic acids with DNases and RNases. The second consists of the selective precipitation of dsRNA using lithium chloride salt solutions (Diaz-Ruiz and Kaper, 1978). Little is known about the yield of the described techniques nor the quality of the purified dsRNAs.

In the current paper, we compare different protocols for the cost-effective extraction and purification of dsRNA from HT115 bacterial cells expressing *dsRNA* constructs of the *Green Fluorescent Protein* (*GFP*) or the *Helicoverpa*-specific gene *Dicer2* (*Dcr2*). Therefore, we assay the impact of three pre-treatments on the efficiency of total RNA extraction with phenol-guanidine. Furthermore, we analyze the quality and quantity of the resulting dsRNA extracts. In addition, we evaluate the applicability of two dsRNA purification approaches on their yield and cost-efficiency. Finally, we demonstrate the useability of thus derived dsRNA for *in vitro* knockdown studies in a lepidopteran cell line.

## 2 Materials and Methods

### 2.1 Gene Sequence Retrieval and Phylogenetic Analysis

The *Helicoverpa armigera Dicer* transcript sequences were identified through the BLAST (Altschul et al., 1990) algorithm by using orthologous genes from closely related species or model organisms as queries and *H. armigera* transcriptome shotgun assemblies (TSAs) as search databases. TSA hits were verified manually on completeness, open reading frames and protein translations via the SnapGene software (Insightful Science) and conserved domains identified through the Conserved Domain Database (Lu et al., 2020). The identified *Helicoverpa armigera* Dicer protein sequences were aligned with known insect proteins through the MUSCLE (Edgar, 2004) algorithm on the MEGA7 (Kumar et al., 2016) platform using default parameters and 200 iterations. The aligned sequences were then exported to IQ-TREE (Nguyen et al., 2015) version 1.6.12 and their phylogenetic relationship was calculated by automatically determining the best substitution model through ModelFinder (Kalyaanamoorthy et al., 2017), performing 10.000 ultrafast bootstraps (Hoang et al., 2018) and carrying out a 1,000 replicates SH-aLRT (Guindon et al., 2010) test. The resulting maximum likelihood tree was uploaded to iTOL (Letunic and Bork, 2021) for rooting and rendering. The identified transcripts and a phylogenetic analysis are presented in the supplementary data (Supplementary Table S1; Supplementary Figure S1).

### 2.2 Vector Construction and Bacterial Transformation

The L4440 vector was digested with the HindIII-HF^®^ (New England BioLabs) DNA restriction enzyme following manufacturer’s instructions. The *Helicoverpa Dicer2* sequence was amplified from a cDNA library of adult female *Helicoverpa armigera* using the Q5^®^ High-Fidelity DNA Polymerase (New England BioLabs) with Q5 high GC enhancer and primers with 5′ overhangs (sequences available in Supplementary Table S2) with a total reaction volume of 25 µL. The PCR was carried out with an initial denaturation at 98°C for 30 s, followed by 35 cycles of 98°C for 10 s, 64.6°C for 30 s and 72°C for 30 s, followed by a final extension at 72°C for 2 min. 20 µL of the PCR product was purified with the GenElute™ PCR Clean-Up Kit (Merck) following manufacturer’s instructions. A Gibson assembly was performed with a 1:2 mass to mass ratio of the digested L4440 vector and the purified PCR products using the NEBuilder^®^ HiFi DNA Assembly Master Mix (New England BioLabs). The assembled vectors were transformed into competent HT115 (DE3) *E. coli* cells. Briefly, 10 µL of assembled vector was added to 100 µL competent cells and carefully mixed by stirring. The cells were incubated on ice for 30 min and then transformed via a heat shock treatment of 42°C for 40 s. The cells were then allowed to grow in 250 µL LB medium for 1.5 h. Hereafter, 150 µL of the transformed bacteria were plated on LB agar plates containing ampicillin (50 μg/ml) and tetracycline (12.5 μg/ml). The plates were incubated at 37°C overnight. Individual colonies were picked and grown in 5 ml LB with ampicillin (50 μg/ml) and tetracycline (12.5 μg/ml) with shaking overnight. Plasmids were extracted from 2 ml of the grown cells using the GenElute™ HP Plasmid Miniprep Kit (Merck) following manufacturer’s instructions. The extracted plasmids were sequenced via sanger sequencing (LGC, Biosearch Technologies) to verify the sequence of the cloned insert.

### 2.3 Bacterial Culturing and Induction of dsRNA Expression

HT115 bacteria containing the L4440-*HaDcr2* or L4440-*GFP* constructs (insert sequences in Supplementary Table S3, L4440-*GFP* assembly described in Vogel, 2020) were plated onto LB plates with ampicillin (50 μg/ml) and tetracycline (12.5 μg/ml) and incubated overnight at 37°C.

Individual colonies were picked and resuspended in 20 µL MilliQ water. 2 µL of the resuspended cells were used in colony PCR reactions using 6.5 µL REDTaq^®^ DNA polymerase (Merck) and 2 µL each of the gene specific forward and reverse primers (10 µM) for a total reaction volume of 12.5 µL. For each colony, PCR reactions were run to verify the bacterial strain and the presence of the correct L4440 plasmid. The bacterial strain identity was established using primers located 5’ upwards and within the T7 polymerase genomic insert (primers HT115 forward and HT115 reverse, respectively). The presence of the L4440 plasmids was assayed using the M13 forward primer and an insert-specific reverse primer (*GFP* reverse or *Dicer2* reverse). All primer sequences are available in Supplementary Table S2. The PCR reactions were run with the following conditions: 96°C for 5 min; then 25 cycles of 96°C for 45 s, 53°C 45 s, 72°C for 1 min; then a final elongation step of 72°C for 3 min. The PCR product sizes were visualized as described in Section 2.6. The agarose gel results are displayed in Supplementary Figure S2 ***.***

Cells from verified colonies were grown in 5 ml selective LB medium with ampicillin (50 μg/ml) and tetracycline (12.5 μg/ml) at 37°C with shaking overnight. 4 ml of the cells were then transferred to 400 ml fresh selective LB medium and incubated again at 37°C with shaking for 4 hours. Hereafter, the optical density (OD) of the cell culture was assayed using a Ultrospec^®^ 10 Cell Density Meter (Biochrom). Once the OD was above 0.4, the bacterial cells were induced with sterile filtered Isopropyl β-d-1-thiogalactopyranoside (IPTG) to a final concentration of 1 mM. Five hours after the induction, the cells were pelleted by centrifugation (4,000 rcf for 10 min at 4°C), they were resuspended in 1 ml Milli-Q^®^ water (MilliporeSigma) (henceforth abbreviated to “MQ”) and transferred to a 15 ml falcon tube.

### 2.4 Bacterial Total RNA Extraction

The collected cells were freeze-thawed 10 times using liquid nitrogen. Next, MQ was added to a final volume of 4 ml (100x concentrated). They were stored at −80°C until further usage.

Four alternative protocols were evaluated to increase the release of RNA from the bacterial cells. The protocols differed in the treatments applied prior to RNA extraction (the pre-treatments). The applied pre-treatments consisted of either (A) the digestion of the bacterial cell wall with lysozyme, (B) the disruption of the bacterial cells via sonication, (C) the lysis of the bacterial cells via heating in QIAzol, or (D) a control protocol where no pre-treatment was applied. The pre-treatments were carried out as follows:

(A) **Lysozyme Digestion**: 100 µL of cells were pelleted by centrifugation (10,000 rcf for 5 min at 4 °C). The supernatant was removed, and the pellet treated with 100 µL of a lysozyme digestion mix consisting of 1.3 µL of 10 mg/ml lysozyme solution (in 50% v/v glycerol), 5.2 µL of 0.5 M EDTA, 13 µL Triton X-100 and 1.3 ml MQ water at 37 °C for 30 min. The digested cells and supernatants were then combined and mixed with 1 ml of QIAzol.
(B) **Sonication**: 100 µL bacterial cell suspension was sonicated on ice for three cycles of 30 s on and 30 s off using the SLPe digital sonifier (Brandson) at 10% amplitude. The sonicated cells were then mixed with 1 ml QIAzol.
(C) **Heating**: 100 µL of bacterial cell suspension was mixed with 1 ml of QIAzol by vortexing for 5 s. The homogeneous mixture was then incubated for 30 min in a 60°C oven.
(D) **Control (no Pre-Treatment)**: 100 µL of bacterial cell suspension was mixed with 1 ml of QIAzol by vortexing for 5 s.

The QIAzol-cell mixtures were then incubated at RT for 5 min. Hereafter, 0.2 ml of chloroform was added and the samples vortexed for 15 s. The samples were left at rest for 10 min and then centrifuged at 12,000 rcf for 15 min at 4°C. After phase separation, the aqueous phase had a volume of approximately 700 µL. To avoid contamination from the interphase, 600 µL of the aqueous phase was taken and transferred to a clean 1.5 ml Eppendorf tube. 500 µL of isopropanol was added and the samples briefly vortexed. Hereafter the samples were left to stand for 7 min, then spun at 12,000 rcf for 10 min at 4°C. The supernatants were removed, and the RNA pellet washed with 1 ml of 70% (v/v) EtOH in RNase-free water. The samples were then once more spun at 7,500 rcf for 5 min at 4 °C. The supernatants were removed, and the RNA pellet was left to dry for 10 min, after which the RNA was resuspended in 180 µL RNase-free water. The absorbance at 260 nm (A_260_) was assessed through a NanoPhotometer^®^ N60 (Implen), and the total RNA concentration estimated using a conversion factor of 40 μg/ml/A_260_. The absorbance measurements are provided in the Supplementary Table S4.

Furthermore, the scalability of the protocol was assessed by extracting the total RNA from 800 ml of induced bacterial culture. The heating pre-treatment was applied. Briefly, the bacteria were pelleted by centrifugation (4,000 rcf for 10 min at 4°C) and resuspended in MQ. The cells were split in two equal volumes. 40 ml of QIAzol was added to each volume and the mixtures vortexed until homogeneous. The mixtures were then incubated in a 60°C water bath for 30 min; 8 ml chloroform was added to each tube and the mixtures briefly vortexed. The combined volume was distributed over three 50 ml centrifuge tubes with sealing cap (Nalgene). Phase separation was allowed to occur for 7 min, then the samples were spun at 12,000 rcf in a Multifuge X1R (Heraeus) for 15 min at 4°C. The supernatants (∼13 ml per tube) were collected in new 50 ml falcon tubes and combined with 9 ml isopropanol. The mixtures were incubated at RT for 7 min then again spun at 12,000 rcf for 15 min at 4 °C. The resulting RNA pellets were washed three times with 5 ml 70% (v/v) EtOH in RNase-free water, pelleting the RNA by centrifuging at 7,500 rcf for 5 min at 4°C between each wash. Finally, the remaining traces of EtOH were removed from the RNA pellets via evaporation and the RNA from all tubes combined and dissolved in 10 ml RNase-free water.

### 2.5 dsRNA Purification

Total RNA extracts were treated for the purification of the dsRNA fraction. Two approaches were employed, enzymatic digestion of DNA and/or ssRNA, and selective precipitation of dsRNA.

#### 2.5.1 Enzymatic Digestion

To digest the ssRNA fraction, the total RNA was treated with RNase A (Thermo Scientific). As RNase A can also cleave dsRNA under low salt conditions (Libonati and Sorrentino, 2001), two buffers were compared in their ability at shielding the dsRNA from degradation. These were (1) a NaCl-based buffer and (2) the TURBO™ DNase digestion buffer (Thermo Scientific), hereafter referred to as “TURBO buffer”.

(1) NaCl Buffer: 1.125 A_260_ units (1 A_260_ unit is defined as the amount of nucleic acid that produces an OD of 1 in 1 ml) of total RNA were diluted to a volume of 90 µL with RNase-free water and mixed with 10 µL of a 10x RNase digestion buffer consisting of 300 mM NaCl, 10 mM Tris-HCl and 5 mM EDTA with neutral pH. The mixture was incubated at 37 °C for 30 min. Hereafter, 1 µL of 10 mg/ml RNase A (Thermo Scientific) was added and the sample incubated at 37°C for an additional hour.
(2) TURBO Buffer: Ahn et al. (2019) demonstrated that the TURBO buffer can be used to purify dsRNA from bacterial total RNA extracts, with the added benefit of allowing a simultaneous DNase digestion. To this end, 1.125 A_260_ units of total RNA were diluted to a volume of 90 µL with RNase-free water and mixed with 10 µL of 10x TURBO™ DNase digestion buffer (Thermo Scientific). The mixture was incubated at 37°C for 30 min. Hereafter, 1 µL of 2 U/µl TURBO™ DNase (Thermo Scientific) and 1 µL of 10 mg/ml RNase A (Thermo Scientific) were added and the sample was incubated at 37°C for an additional hour.

The RNA was then extracted from the digested samples following the QIAzol extraction protocol, with 500 µL QIAzol. This resulted in a 350 µL water phase. The RNA was precipitated with 350 µL isopropanol and washed with 0.5 ml 70% (v/v) EtOH in RNase-free water. The purified dsRNA was then resuspended in 50 µL RNase-free water.

#### 2.5.2 Selective Precipitation

The dsRNA fraction was purified via selective precipitation in a LiCl solution. This protocol is hereafter referred to as “LiCl precipitation”. For this protocol, 1.125 A_260_ units of total RNA were diluted to a final volume of 150 µL using RNase-free water. 1/3^rd^ volume of 8M ice-chilled RNase-free LiCl (Merck) was added to the sample and mixed for a final concentration of 2 M LiCl. The mix was incubated at −20°C for 30 min, then centrifuged at 16,000 rcf for 30 min at 4°C. The supernatant was carried over to a new clean tube and combined with ½ volume of 8 M RNase-free ice-chilled LiCl to a final concentration of 4 M. The samples were incubated at −20°C overnight, then once more centrifuged at 16,000 rcf for 30 min at 4°C. The supernatants were discarded, and the dsRNA pellet washed with 500 µL 70% (v/v) EtOH in RNase-free water. The samples were again centrifuged at 16,000 rcf for 5 min at 4°C. The supernatants were removed, and the pellet allowed to dry. The dsRNA was then resuspended in 50 µL RNase-free water.

The scalability of the LiCl precipitation protocol was assessed on 5 ml of RNA extracted from 400 ml induced bacteria. The given protocol was followed until the dsRNA pellet was obtained. The pellet was washed thrice with 5 ml 70% (v/v) EtOH in RNase-free water and the sample spun at 4,000 rcf for 10 min at 4°C. The supernatant was removed, and the pellet allowed to dry. The dsRNA was then resuspended in 1 ml RNase-free water.

The A_260_ of the purified dsRNA solutions was assessed with a NanoPhotometer^®^ N60 (Implen). The absorbance measurements are provided in Supplementary Table S6. The dsRNA concentration was then calculated using a conversion factor of 46 μg/ml/A_260_, as determined by Strezsak et al. (2021).

### 2.6 Agarose Gel Electrophoresis

To visualize the size of the PCR products, or the size and integrity of dsRNA strands in RNA extracts or purifications; the nucleic acids were run on a non-denaturing 1% agarose gel in TAE buffer at 120V for half to 2 hours (precise timing given in figure descriptions). The gel was stained with the GelRed^®^ (Biotium) nucleic acid stain and imaged on a ProXima 2000 series platform (Isogen).

### 2.7 *In vitro* dsRNA Synthesis

L4440 plasmids containing the target constructs were used as templates for PCR reactions using the REDTaq^®^ DNA polymerase. For each reaction, 2.5 ng of plasmid was combined with 6.25 µL REDTaq^®^ and 0.5 µL of forward and reverse primers with T7 overhangs (10 µM) with a final volume of 12.5 µL. The primer sequences are shown in Supplementary Table S2 ***.***

The PCR was run as follows: 98°C for 5 min, followed by 5 cycles of 98°C for 45 s, 55°C for 45 s and 72°C for 1 min. This was followed by 35 cycles of 98°C for 45 s, 60°C for 45 s and 72°C for 1 min. Finally, the reaction mixture was incubated at 72°C for 2 min and then stored at 4°C. 8 µL of the PCR products were used as substrates for MEGAscript™ T7 transcription reactions (Thermo Scientific). dsRNA was produced and purified following manufacturer’s instructions.

### 2.8 Knockdown in a Cell Line

*Helicoverpa zea* derived RP-*Hz*GUT-AW1 cells (Goodman et al., 2004) were kept in Ex-Cell^®^ 420 (Merck) medium supplemented with 10% fetal bovine serum (Sigma-Aldrich), 100 U/ml penicillin (Gibco), 100 mg/ml streptomycin (Life Technologies) and 0.25 μg/ml amphotericin B (Sigma-Aldrich). The cells were grown at 27.5°C and subpassaged 1/3 at 90% confluency.

To assess viability and concentration, 10 µL of scraped RP-*Hz*GUT-AW1 cells were mixed 1:1 with 0.4% Trypan Blue (Sigma-Aldrich) and loaded onto a cell counting slide. Cell viability and concentration were measured with the Bio Rad TC20™ Automated Cell Counter.

Cells with a viability above 95% were plated at a density of 2.5 × 10^5^ cells/well in 24-well plates. They were left to attach for 3 hours, after which time they were washed with 200 µL of non-supplemented Ex-Cell^®^ 420 (Merck) medium and then treated with the transfection medium.

The transfection medium was prepared by combining 450 ng dsRNA (2.5 μg/ml condition) or 90 ng dsRNA (0.5 μg/ml condition) with 90 µL non-supplemented Ex-Cell^®^ 420 (Merck) and letting the mixture incubate at room temperature for 30 min. In parallel, 90 µL of non-supplemented Ex-Cell^®^ 420 (Merck) was mixed with 3.7 µL Escort IV (Sigma-Aldrich) transfection reagent and likewise left to incubate at RT for 30 min. After the incubation period, the two volumes were combined and the lipoplexes allowed to form for an additional 20 min at RT. The resulting 180 µL transfection mixture was then added directly to the cells. The cells were placed overnight at 27.5°C, after which the transfection medium and non-adherent cells were removed and replaced by 500 µL of supplemented Ex-Cell^®^ 420 (Merck) medium. The cells were grown for one more day after which they were loosened by pipetting and collected in 1.5 ml tubes. The cell viability and concentration were assayed as described above. The collected RP-*Hz*GUT-AW1 cells were pelleted by spinning for 10 min at 1,000 rcf and 4 °C. The supernatant was removed, and the cells resuspended in 10 µL Milli-Q^®^ water. The cells were then stored at −80°C.

### 2.9 RP-*Hz*GUT-AW1 RNA Extraction and cDNA Synthesis

Total RNA was extracted from the frozen RP-*Hz*GUT-AW1 cells with the RNeasy Lipid Tissue Mini Kit (Qiagen) following manufacturer’s instructions. The DNase digestion step was included to remove potential genomic DNA contamination. Quality and quantity of the extracted RNA were assessed with a NanoPhotometer^®^ N60 (Implen) using a conversion factor of 40 μg/ml/A_260_. cDNA was synthetized from 400 ng RNA in a 10 µL reaction using the PrimeScript First Strand cDNA Synthesis Kit (TaKaRa) following manufacturer’s instructions.

### 2.10 Quantitative Real Time PCR

Target gene expression was assessed through a QuantStudio™ 3 Real-Time PCR System (Thermo Scientific). All PCR reactions were carried out in duplicate with 5 µL Fast SYBR™ Green Master Mix (Thermo Scientific), 0.375 µL 10 µM of the forward and reverse primers, and 4.25 µL cDNA (synthetized from 6.67 ng RNA). All qPCR primers used are presented in Supplementary Table S2.

Primer efficiency was determined through relative standard curves with 5x serial dilutions. Specificity and primer-dimer formation were assessed through melting curves. Only primers with efficiencies between 90 and 110% with single melt peaks were used. Gene expression was determined through the delta-delta Ct method (Livak and Schmittgen, 2001). The *Helicoverpa zea* housekeeping genes actin (GenBank: AF286061.1) and arginine kinase (GenBank: HM068068.1) were selected for normalization from a list of candidates through the geNorm algorithm in the NormqPCR package (Perkins et al., 2012).

### 2.11 Statistical Analysis

Data analysis was carried out using the GraphPad Prism 8 software. To assay for significant differences in the concentration of RNA extracts dependent on the pre-treatment applied and the dsRNA construct used, a two-way ANOVA was performed. A *post hoc* Tukey’s multiple comparisons test was carried out to identify which pre-treatments yielded significantly different RNA concentrations. Next, the impact of three independent variables (pre-treatment, extraction method and dsRNA construct) was assessed on: (1) the fraction of dsRNA in the total RNA samples, and on (2) the final dsRNA production yield. To this end, repeated measure two-way ANOVA tests were performed using: (1) the percentage of A_260_ units retained after purification (Table 1) or (2) the calculated dsRNA yields (Table 2), respectively. No sphericity was assumed, and *p*-values adjusted with the Geisser-Greenhouse method. For both tests, *post hoc* Tukey’s multiple comparisons tests were used to identify which pre-treatments significantly differed. The effect of the type of dsRNA used for transfection of RP-*Hz*GUT-AW1 on the viability and cell count was determined with a one-way ANOVA. The significance of the resulting gene knockdowns was calculated through multiple *t*-tests, with alpha = 0.05. Each knock down condition was analyzed individually, without assuming a consistent SD. To assay possible differences in the knockdown efficiencies of the tested dsRNA types, a one-way ANOVA with Brown-Forsythe and Welch tests was applied. All analysis results are provided in the text or in the Supplementary Tables S5, 7–9.

**TABLE 1:** Percentage of A_260_ units retained after treatment of total RNA (%A_260 dsRNA_) from *dsGFP* or *dsDcr2* producing bacteria for all combinations of pre-treatments and purification methods tested.

%A_260 dsRNA_ *GFP*	Purification method			Average per pre-treatment
	LiCl precipitation	NaCl buffer	TURBO buffer	
Pre-treatment				
Lysozyme	14.1	16.9	16.8	15.9
Sonication	20.9	20.6	20.2	20.6
Heating	18.2	18.0	16.4	17.5
Control	21.6	20.9	20.6	21.0
Average per purification method	18.7	19.1	18.5	
**%A_260_ dsRNA *Dcr2***				
Pre-treatment				
Lysozyme	11.4	13.5	12.9	12.6
Sonication	17.5	17.9	18.4	17.9
Heating	15.4	13.9	16.2	15.2
Control	18.0	16.8	17.3	17.4
Average per purification method	15.6	15.5	16.2	

**TABLE 2:** dsRNA production yield (µg ds*GFP* or ds*Dcr2* per ml bacterial culture) for all combinations of pre-treatments and purification methods tested.

µg/ml ds*GFP*	Purification method			Average per pre-treatment
	LiCl precipitation	NaCl buffer	TURBO buffer	
Pre-treatment				
Lysozyme	3.19	3.51	3.48	3.39
Sonication	7.98	8.06	8.09	8.04
Heating	5.56	4.82	4.38	4.92
Control	2.69	2.51	2.48	2.56
Average per purification method	4.86	4.73	4.61	
**μg/ml ds*Dcr2***				
Pre-treatment				
Lysozyme	3.71	4.63	4.42	4.25
Sonication	9.21	9.08	9.36	9.22
Heating	6.30	5.68	6.60	6.19
Control	3.23	3.10	3.20	3.18
Average per purification method	5.61	5.62	5.89	

## 3 Results

Bacterial systems allow the production of complex organic molecules in a highly scalable and cost-effective manner. As such they are attractive candidates for the production at medium to large-scale of dsRNA constructs in a laboratory setting. Up to date, bacterially produced dsRNA is rarely applied for *in vitro* studies, partly due to as yet inefficient or unreliable extraction techniques, and partly due to the lack of trust in the purity and effectiveness of bacteria-derived dsRNA. Some major obstacles that need to be overcome are: (1) the efficient extraction and (2) the purification of the dsRNA from the bacterial cells, including the removal of biological contaminants. The protocols developed for this purpose must be (3) scalable while maintaining the quality of the final product. The produced dsRNA should (4) not be cytotoxic and (5) perform on par with other commercially available alternatives. Based on the results presented in this paper we demonstrate that the applied methods satisfy each of these criteria.

### 3.1 Pre-Treatments Reliably Improve Total RNA Extraction Efficiency

**FIGURE 1:**
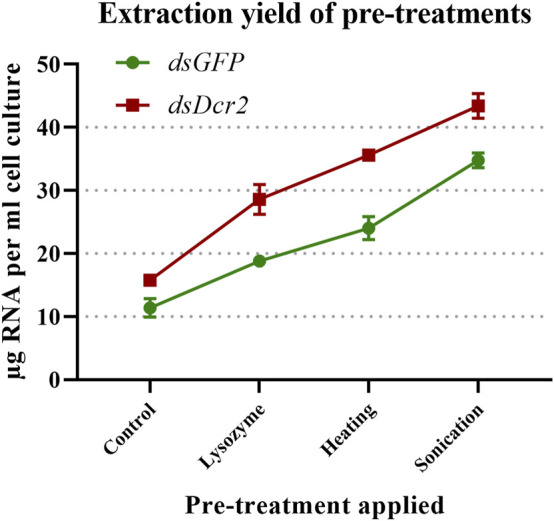
Impact on total RNA extraction efficiency of three pre-treatments. Concentrated bacterial cells expressing two dsRNA constructs (*dsGFP* or *dsDcr2*) were freeze-thawed ten times and then subjected to cell-disruption treatments. The total RNA was then extracted through a phenol/guanidine-based method and the concentration measured through a spectrophotometer. Four replicates were tested for each condition (32 samples in total). Symbols indicate the mean, error bars the standard deviation. All pre-treatments significantly (*p* < 0.0001, two-way ANOVA with Tukey’s multiple comparisons test) increased the extraction yields compared to the control method and differed between each other.

A major challenge to the production of dsRNA from bacterial systems is the efficient release of the dsRNA from the cells without affecting its integrity. A commonly applied technique is the use of phenol/guanidine-based lysis reagents. While efficient for most eukaryotic cells and tissues, this method often results in low yields due to the presence of a rigid bacterial cell wall. To overcome this issue, we assayed how the total RNA extraction yield of a control protocol was affected when applying three pre-treatments prior to extraction. The control protocol consisted of an incubation in a phenol/guanidine-based lysis reagent for 5 minutes at room temperature. The designator “control” was chosen as this treatment is commonly found in phenol/guanidine-based protocols, and thus functioned as a baseline through which the impact of additional pre-treatments could be established. The pre-treatments were: (A) digestion of the bacterial cell wall with lysozyme, (B) sonication of the bacterial cells, and (C) heating of the cells in lysis reagent. For this purpose, 100x concentrated and freeze-thawed bacterial cells containing the L4440-*HaDcr2* or L4440-*GFP* constructs were aliquoted in 100 µL volumes and subjected to the described pre-treatments. Per condition four replicates were included in this analysis. Total RNA was then extracted from the pre-treated and control cells. The A_260_ of the total RNA was measured (Supplementary Table S4), the concentration calculated with a conversion factor of 40 μg/ml/A_260_ and the RNA was run on an agarose gel. To find whether the pre-treatments affected the extraction efficiency, a two-way ANOVA was applied (statistics collected in Supplementary Table S5) on the collected *dsGFP* and *dsDcr2* data. The results indicated that the pre-treatments as well as the identity of the dsRNA construct significantly affected the extraction efficiency (*p* < 0.0001). A *post hoc* Tukey’s multiple comparisons test shows that all pre-treatments significantly (*p* < 0.0001) differed from each other and the control (Figure 1). Sonication resulted in the highest increase relative to the control, with an average of 2.9-fold, followed by heating with a 2.2-fold increase and digestion with a 1.8-fold increase.

**FIGURE 2:**
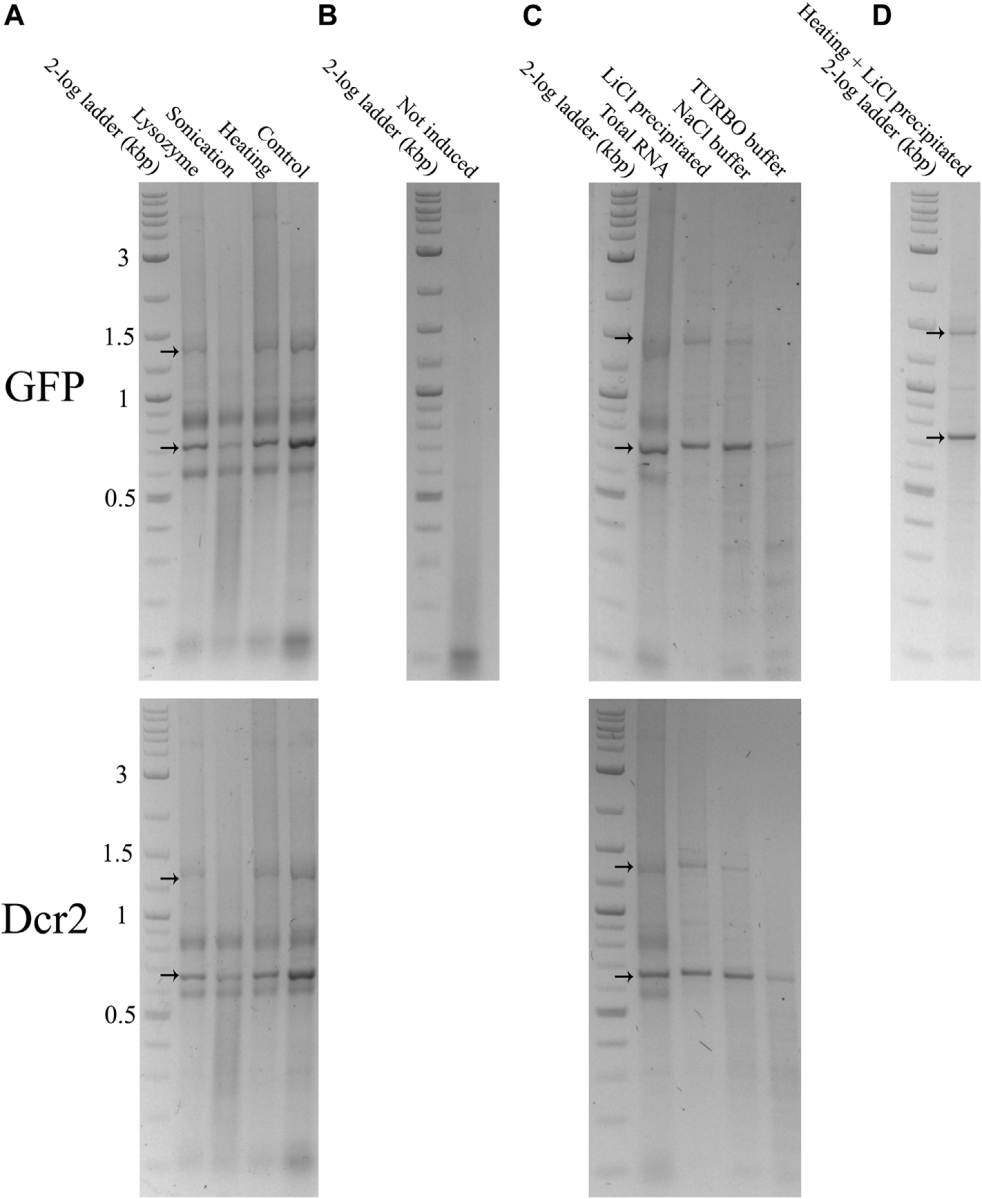
Total and purified RNA extracted from HT115 (DE3) bacteria transformed with a L4440-*GFP* (upper gels) or L4440-*Ha*Dcr2 (lower gels) expression construct was loaded into 1% agarose gels in TAE buffer. The RNA was separated by size through electrophoresis at 120V for 2 h. The nucleic acids were imaged with the GelRed^®^ (Biotium) stain under UV light. Arrows indicate the *dsGFP* and *dsDcr2* bands at the ∼700 bp and ∼650 bp positions of the DNA ladder and the persistent secondary structure dsRNA band at ∼1,400 bp and ∼1,300 bp respectively. **(A)**: 400 ng total RNA extracted from induced bacteria treated with a control protocol or three pre-treatments. **(B)**: 400 ng total RNA extracted using the heating pre-treatment from non-induced bacteria. **(C)**: Comparison between 400 ng total RNA extracted from induced bacteria with the heating pre-treatment and dsRNA purified from 400 ng total RNA through three purification methods. The band intensities indicate the efficiency of the applied dsRNA purification protocols. Thus, the “Heating” lane in panel A is equal to the “Total RNA” lane in panel **(C)**. **(D)**: 92 ng dsRNA purified from 400 ml of induced bacterial culture using the heating pre-treatment and LiCl precipitation.

400 ng of total RNA from *dsGFP* or *dsDcr2* expressing bacteria extracted through each treatment were run on a 1% agarose gel (Figure 2A). As a negative control, total RNA from non-induced cells transformed with the *L4440-GFP* construct and extracted via the heating pre-treatment was likewise run on a 1% agarose gel (Figure 2B). The *dsGFP* and *dsDcr2* constructs with an expected size of 775 bp and 712 bp respectively, were at ∼700 bp and ∼650 bp due to the higher electrophoretic mobility of dsRNA compared to the dsDNAs represented in the ladder. As expected, the *dsGFP* band was missing in the negative control sample. For all treatments and constructs the dsRNA band was present at the expected distance. The intensity of the dsRNA band varied between treatments. The band intensity is dependent on (A) the fraction dsRNA in the total RNA and (B) the degree of degradation of the dsRNA. Total RNA extracted through the control method displayed the strongest dsRNA band, indicating a large fraction of dsRNA; while the RNA extracted from sonicated cells displayed the weakest band with a strong smear underneath the construct, suggesting band degradation.

### 3.2 Purification Protocols Yield Enriched and Intact dsRNA

The total RNA extracted from the bacteria must be treated to isolate the dsRNA fraction and remove undesired bacterial RNAs. In this study we compared the efficiency and practicality of two distinct purification approaches. The first consisted in the selective precipitation of dsRNA through lithium chloride. This method will be referred to as “LiCl precipitation” throughout the text. The second approach was based on the enzymatic digestion of ssRNA with RNase A. To this end, two nucleic acid digestion buffers were tested: a NaCl-based buffer (henceforth called “NaCl buffer”), and the commercial TURBO™ DNase digestion buffer (henceforth called “TURBO buffer”). As the TURBO buffer was designed for the TURBO™ DNase, a concurrent digestion of the DNA fraction was caried out for this method. For all three purification methods, the *dsGFP* and *dsDcr2* fractions were purified from 1.125 A_260_ units total RNA of one sample obtained through each of the extraction treatments (24 samples total). The absorbance of the purified dsRNA (available in Supplementary Table S6) was used to calculate the percentage of A_260_ units retained after purification (Table 1). For simplicity, this percentage (which serves as a proxy for the fraction dsRNA in the total RNA extracts) will be referred to as “%A_260 dsRNA_” in the text.

**FIGURE 3:**
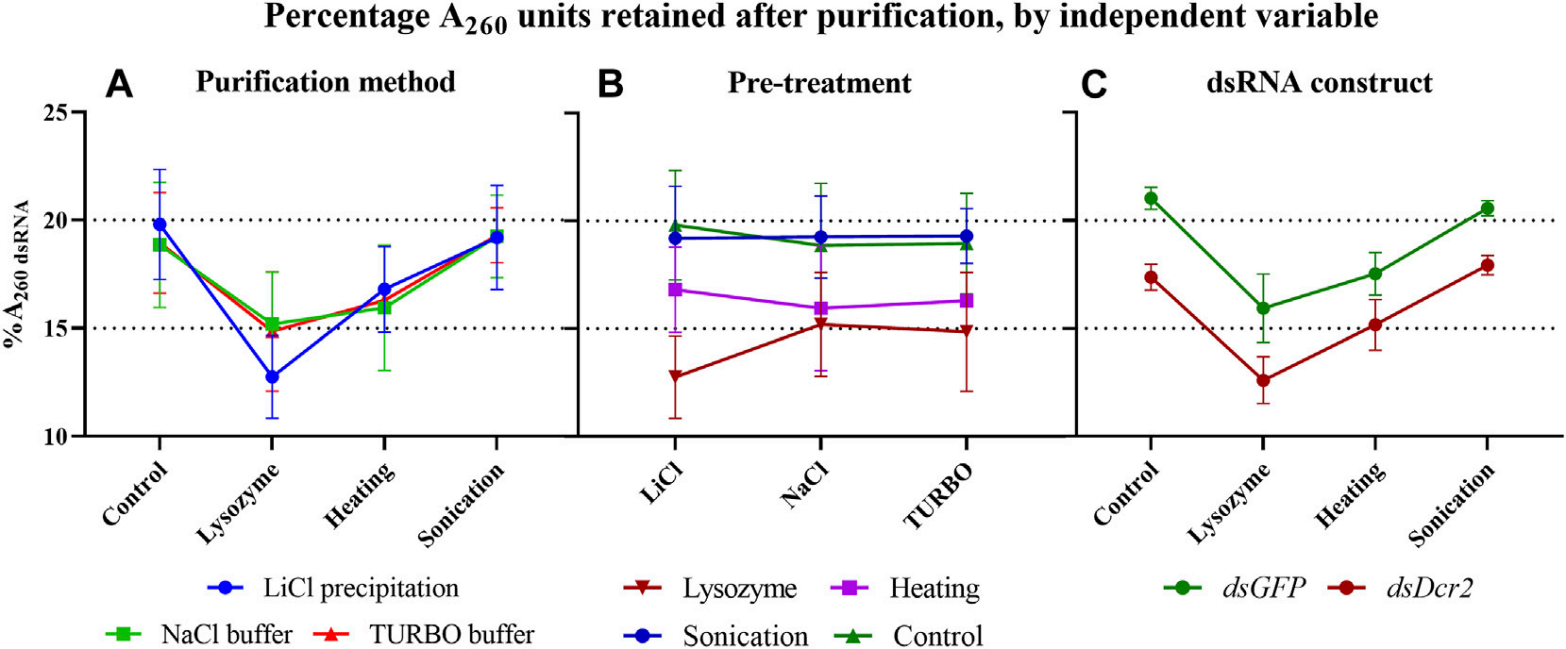
In these graphs, the data of Table 1 are plotted to highlight the impact of the three independent variables (purification method, pre-treatment, and dsRNA construct) on the percentage of A_260_ units retained following dsRNA purification (%A_260 dsRNA_). When the data are sorted on the y-axis by one variable (pre-treatment for panel **(A,C)**, or purification method for panel **(B)** and pooled as replicates by a second variable (dsRNA construct for panel A and B, or purification method for panel **(C)**, the effect of the third variable can be visualized. Symbols indicate the mean, error bars the standard deviation. **(A)**: The purification methods have no significant impact (*p* = 0.9945) on the %A_260 dsRNA_. **(B)**: The pre-treatments have a major effect (*p* = 0.0007) on the %A_260 dsRNA_. **(C)**: The *GFP* construct results in a higher %A_260 dsRNA_ than the *Dicer2* construct (*p* < 0.0001). Statistical analysis performed with a repeated measure two-way ANOVA.

A repeated measure two-way ANOVA (not assuming sphericity) analysis indicates that the purification methods had no impact on the %A_260 dsRNA_ (*p* = 0.9945, Figure 3A), while finding a significant dependence on the extraction pre-treatments (p = 0.0007, Figure 3B). Furthermore, the analysis indicates that the dsRNA construct also had a highly significant impact (p < 0.0001, Figure 3C). A *post hoc* Tukey test shows that the sonication pre-treatment and the no pre-treatment control differed significantly from the lysozyme and heating pre-treatments (p ≤ 0.0040), with no significant difference within the two pairs (Supplementary Table S7; Figure 3B). The data presented in Table 1 show that the control and sonication treatments yielded the largest fraction (∼20.8 and ∼17.7%A_260 dsRNA_ for *dsGFP* and *dsDcr2*, respectively) of dsRNA in the total RNA extracts. The data also indicate that the selective precipitation of dsRNA with lithium chloride and the enzymatic digestion of nucleic acids yielded comparable purification efficiencies (∼18.8 and ∼15.8%A_260 dsRNA_ for *dsGFP* and *dsDcr2*, respectively).

**FIGURE 4:**
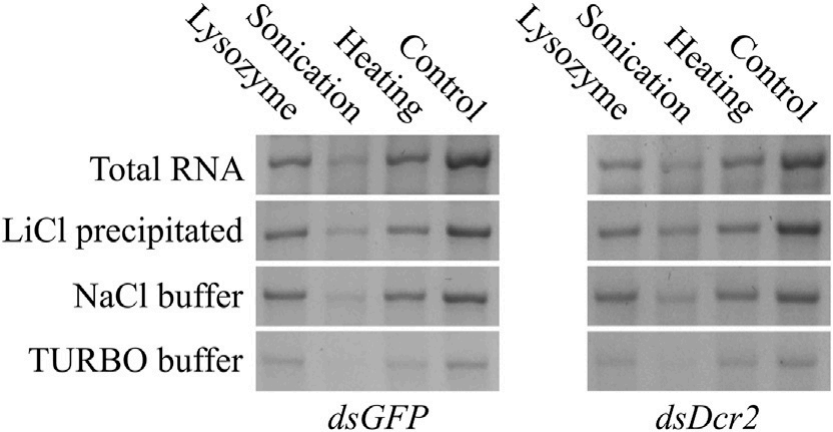
Comparison of *dsGFP* (∼700 bp) and *dsDcr2* (∼650 bp) bands of total and purified RNA. For each condition, 400 ng total RNA or dsRNA purified from 400 ng total RNA were loaded onto a 1% agarose gel in TAE buffer. The RNA was separated by size through agarose gel electrophoresis at 120V for 2 h. The nucleic acids were imaged with the GelRed® (Biotium) stain under UV light.

The purified dsRNA fractions were analyzed through agarose gel electrophoresis to verify the removal of bacterial ssRNA or DNA (Figure 2C) and assess the integrity and intensity of the purified dsRNA band (Figure 4). The data show that the purification via LiCl precipitation or RNase A digestion with the NaCl buffer did not affect the integrity of the dsRNA bands. In contrast, RNase A digestion with the TURBO buffer resulted in a visibly lower band intensity, suggesting degradation.

**FIGURE 5:**
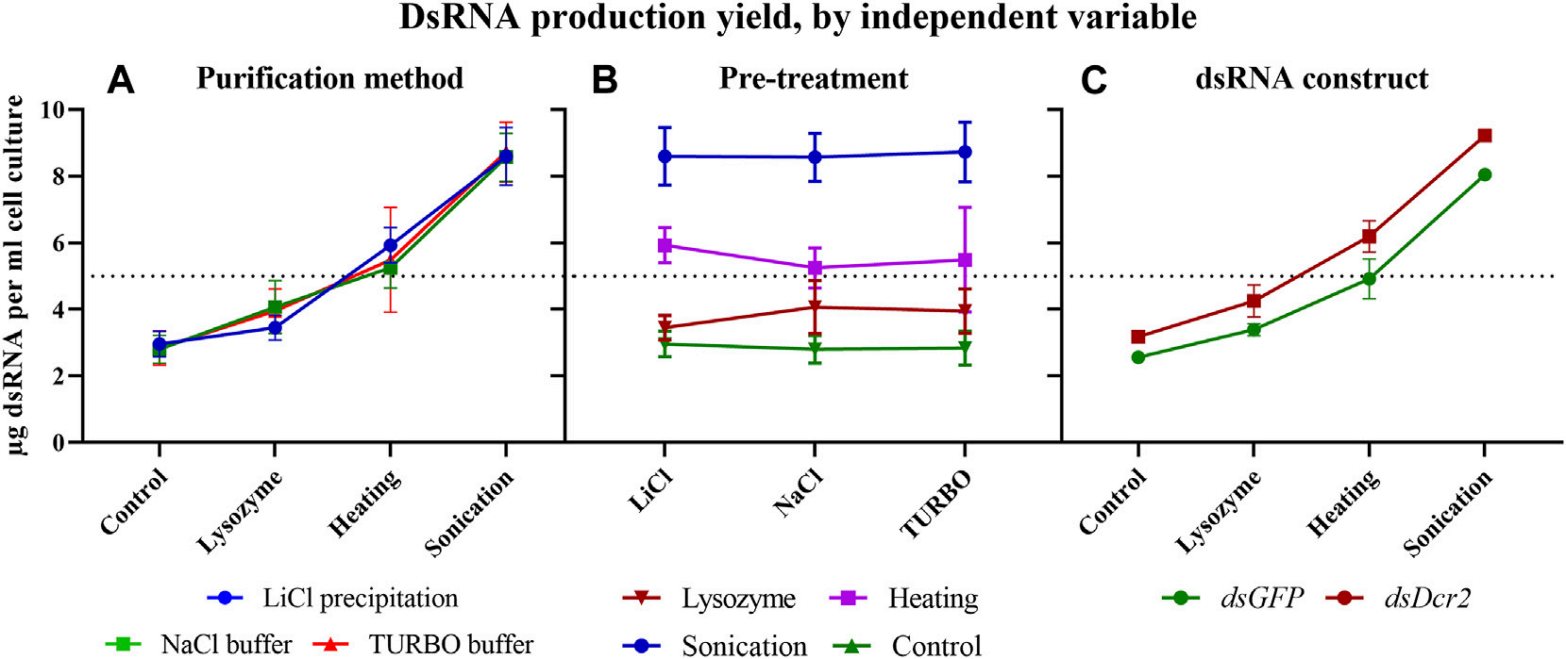
The production yield of dsRNA per ml of bacterial culture is dependent on the pre-treatments applied and the dsRNA construct. The data of Table 2 were sorted as described in Figure 4 to visualize the impact of the experimental variables on dsRNA yield. Symbols indicate the mean, error bars the standard deviation. **(A)**: Purification methods did not impact the final production yield of dsRNA per ml cell culture (*p* = 0.9935). **(B)**: The pre-treatments had a major effect on the final production yield of dsRNA per ml cell culture (*p* < 0.0001). **(C)**: The dsRNA construct had an impact on the final production yield of dsRNA per ml cell culture (*p* = 0.0002). Statistical analysis was performed with a repeated measure two-way ANOVA.

Starting from the absorbance data of the purified dsRNA and total RNA, we can calculate the amount of purified dsRNA per milliliter of bacterial cell culture for each combination of pre-treatments and purification methods (Table 2). The amount of purified dsRNA was calculated using a conversion factor of 46 μg/ml/A_260_ (Strezsak et al., 2021). A repeated measure two-way ANOVA (not assuming sphericity) analysis once more indicated that the purification method had no impact on the final production yield of dsRNA per ml cell culture (*p* = 0.9935). The most determining factor was the pre-treatment applied (94% of variation, *p* < 0.0001), although the dsRNA construct also had a significant impact (5% of variation, *p* = 0.0002). A *post hoc* Tukey’s multiple comparisons test confirmed that all pre-treatments significantly differed between each other (*p* ≤ 0.0086). The full statistical results are given in Supplementary Table S6. The data indicate that the sonication pre-treatment yielded the largest amount of dsRNA per volume unit of bacterial cell culture, with an average 3.0-fold increase compared to the control for both dsRNA constructs. The second largest amount was obtained through the heating pre-treatment with a 1.9-fold increase (Figure 5).

### 3.3 dsRNA Production Protocols Are Scalable

A major advantage of the studied dsRNA extraction and purification protocols is their high scalability. To test whether the dsRNA production yields are independent from the bacterial sample size, *dsGFP* was extracted from 400 ml of induced bacterial culture using the heating pre-treatment and purifying the dsRNA through LiCl precipitation. This procedure yielded a total quantity of 1.67 mg dsRNA, or 4.18 µg dsRNA per ml cell culture. The latter value is comparable to the calculated yield of 5.56 μg/ml with smaller volume purifications (10 ml bacterial culture). The dsRNA band integrity and purity were also maintained (Figure 2D).

### 3.4 Bacteria-Derived dsRNA is Not Cytotoxic to Cultured Cells

**FIGURE 6:**
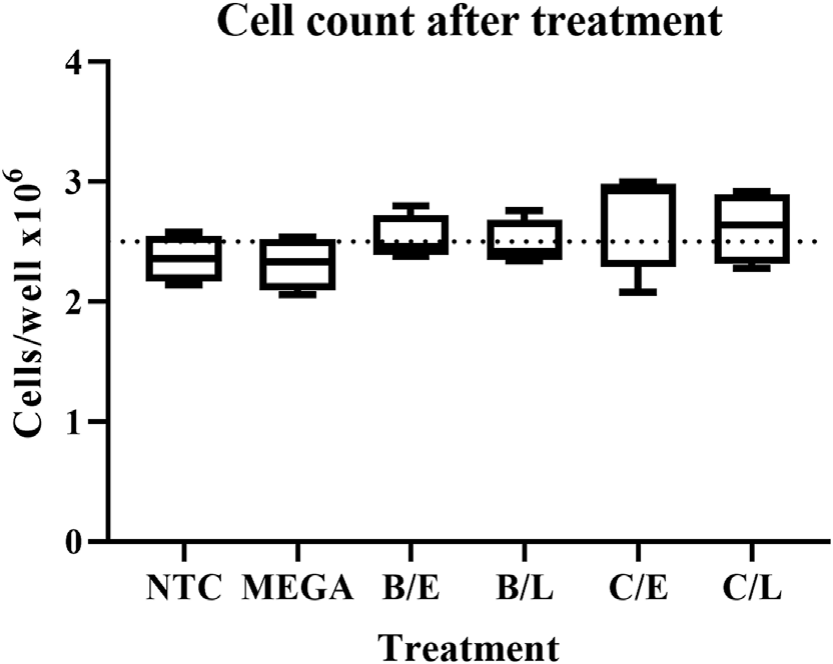
Effect on adherence and growth of RP-*Hz*GUT-AW1 cells of transfection with dsRNA produced *in vitro* or in bacteria. Cells were placed in 24-well plates at > 95% viability and treated with 2.5 μg/ml lipoplexed *dsGFP* or empty lipoplexes overnight. The transfection medium and loose cells were then removed and replaced by complete cell medium. The cells were left to grow for one more day, after which they were collected, and their concentration measured. Four replicates were tested for each condition. Boxplots with min to max whiskers. Dotted line indicates the seeding levels of the cells before treatment. No significant differences were observed (*p* > 0.05) when applying one-way ANOVA analyses. NTC = no-template control consisting of empty lipoplexes; MEGA = dsRNA produced *in vitro* by using the MEGAscript kit; B = sonication pre-treatment; C = heating pre-treatment; E = RNase A digestion in NaCl buffer; L = LiCl precipitation.

A major concern over the useability of bacteria-derived dsRNA in cell line experiments is the impact on cell fitness of possible chemical or biological contaminants. To exclude this possibility, RP-*Hz*GUT-AW1 cells were treated with 2.5 μg/ml *in vitro* or *in vivo* produced *dsGFP* constructs. The cells were then assayed for their viability through an automated cell counter. As the transfection protocol involves a washing step, whereby cells that detached due to possible cytotoxic effects would have been removed, the cell concentration was also assayed, and functions as a measurement of the cell adherence and growth ability. Qualitative and quantitative assays indicated that dsRNAs obtained from sonicated or heated cells and purified through lithium chloride precipitation or digestion by RNase A in the NaCl buffer, had the highest yield. Because of this, the experiment was carried out for all combinations of these methods. One-way ANOVA analyses of viable or adherent cell counting show that there was no significant difference between the conditions tested, neither in terms of cell viability (*p* = 0.1359) nor adherence or growth (*p* = 0.2970, Figure 6).

### 3.5 Bacteria-Derived dsRNA is Applicable for Cellular Knockdown Experiments

**FIGURE 7:**
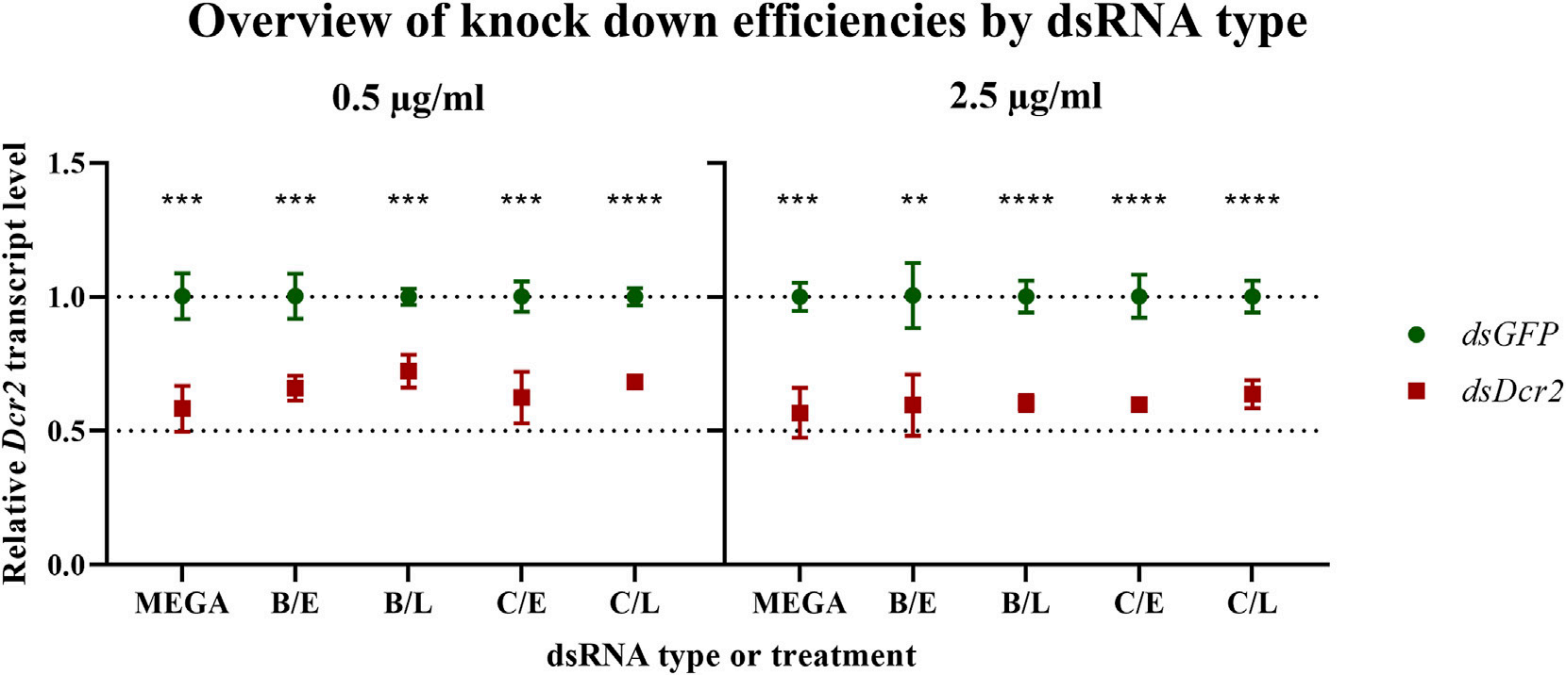
Bacteria-derived dsRNA is effective at inducing a knockdown in the RP-*Hz*GUT-AW1 cell line. Bacteria-derived or MEGAscript produced dsRNA targeting *GFP* or the *Helicoverpa Dicer2* gene was transfected into these gut-derived cells of *Helicoverpa zea*. Transcript levels were assessed 1 day after treatment. Symbols indicate the mean, error bars the standard deviation. Four replicates were tested per condition. All experimental treatments yielded significant knockdowns as assessed through multiple unpaired parametric *t*-test (** *p* ≤ 0.01; *** *p* ≤ 0.001; **** *p* ≤ 0.0001). MEGA = dsRNA produced *in vitro* by using the MEGAscript kit; B = sonication pre-treatment; C = heating pre-treatment; E = NaCl digested dsRNA; L = LiCl precipitated dsRNA.

A major use of dsRNA is the induction of targeted knockdowns through RNA interference. In insects, this process displays a considerable variety of efficiency, with some orders, such as Lepidoptera, requiring high doses of dsRNA for eliciting a significant silencing effect (Shukla et al., 2016). The proposed method for the production of dsRNA in the order of milligrams is particularly interesting for these less sensitive species. For this reason, the ability of bacteria-derived dsRNA at inducing knockdowns was tested in a lepidopteran gut-derived insect cell line. 2.5 × 10^5^ RP-*Hz*GUT-AW1 cells were treated with either 0.5 or 2.5 μg/ml dsRNA complexed with Escort IV transfection reagent overnight. The transfection medium and non-adhering cells were removed and replaced with complete growth medium. One day after treatment the cells were collected and the *Dicer2* transcript levels assayed through quantitative RT-PCR (qRT-PCR) (Figure 7).

For each dsRNA type and concentration, the qRT-PCR ddCt data were normalized against the mean of the control samples and analyzed using multiple *t*-tests. The analysis shows that all dsRNA treatments resulted in significant knockdowns (statistics collected in Supplementary Table S9). Next, Brown-Forsythe and Welch ANOVA tests were conducted on the *dsDcr2* ddCt data for each dsRNA concentration. However, no significant difference among the means of the dsRNA types was observed (for 0.5 μg/ml *p* = 0.1056 and *p* = 0.2355; for 2.5 μg/ml *p* = 0.7495 and *p* = 0.7270). These results indicate that the bacteria-derived dsRNA was equally as effective at inducing target gene knockdown as the *in vitro* produced dsRNA, for both concentrations tested.

## 4 Discussion

The HT115 (DE3) bacterial system in conjunction with the L4440 expression plasmid is a valuable and reliable tool for inducing RNAi in invertebrate species (Timmons et al., 2001). It enables researchers to conduct otherwise prohibitively expensive experiments, requiring manageable development cost and time. Up to date, the system has mainly been applied for *in vivo* feeding studies through bmRNAi (Goodfellow et al., 2019; Taning et al., 2020). In this study, we sought to optimize the isolation of dsRNA from the bacterial production system and demonstrate the usability of thus derived nucleic acids for *in vitro* and *in vivo* knockdown experiments.

### 4.1 Pre-Treatments Reliably Improve Total RNA Extraction Efficiency

One of the limiting factors in the extraction of dsRNA produced in bacteria is the presence of the bacterial cell wall, which hinders the release of dsRNA from the cytoplasm. Two techniques applied to disrupt the cell wall are heating and sonication of the bacterial cells. The heating pre-treatment causes denaturation of organic polymers and disrupts the cell membrane, increasing the porosity of the bacteria and facilitating dsRNA release. The heating treatment can be applied before or after the addition of phenol/guanidine-based lysis reagents. When applied before, bacterial cells are heated to 95–100°C for 10–100 min (Kim et al., 2015; Vatanparast and Kim, 2017; Ahn et al., 2019). This process was shown to greatly increase extraction yields of total RNA from the cells. If the heating is carried out after the addition of the lysis reagent, the sample is heated to 60–70°C for 10–30 min (Sarathi et al., 2008; Ahn et al., 2019). A single heating step after the addition of the lysis reagent is to be preferred, due to possible degradation of the dsRNA in a high-temperature environment rich in polyvalent ions (Ai et al., 2018) and the possibly increased dsRNase activity of bacterial single-stranded RNases under non-physiological conditions (Grünberg et al., 2021). The sonication treatment causes shearing of the bacterial cells through acoustic cavitation. BmRNAi experiments show that sonication increases phenotypic effects, including the insecticidal activity of HT115 cells expressing dsRNA targeting vital genes (Kim et al., 2015; Vatanparast and Kim, 2017; Ahn et al., 2019). The authors hypothesized that this observed increase was due to the improved cellular release. This hypothesis conflicts with typically low RNAi efficiencies observed when feeding naked dsRNA to the animals (Terenius et al., 2011; Lim et al., 2016; Xu et al., 2016). An alternative explanation could be found in the fragmented nature of sonicated dsRNA. A hampered endosomal escape is thought to be one of the limiting factors in RNAi efficiency in Lepidoptera. While tissues and cells of these species promptly take up dsRNA from the environment, the substrate accumulates in acidic endocytic compartments but is never processed into siRNAs (Shukla et al., 2016; Yoon et al., 2017). Diversifying the size distribution of the delivered dsRNA fragments could improve endosomal escape and thus RNAi efficiency.

The data presented in this paper prove for the first time with statistical significance the increase of RNA extraction yield brought forth by three pre-treatments (*p* < 0.0001, Supplementary Table S5). We showed that sonication was the most effective at releasing RNA from the cells (Figure 1), albeit at the cost of reduced dsRNA band integrity (Figure 4; Figure 2A), with a 2.9-fold increased yield compared to a control condition. We also demonstrated that heating the cells in lysis reagent increased the yield by 2.2-fold compared to a control condition while maintaining band integrity. Both treatments proved more effective than an enzymatic digestion of the bacterial cell wall using lysozyme. Notably, while increasing the total amount of RNA extracted per volume of bacterial cells, the heating and lysozyme pre-treatments seemed to decrease the relative fraction of dsRNA, as evidenced by the lower band intensity when a fixed amount of total RNA was separated by gel electrophoresis (Figure 2A) and by the lower residue (%A_260 dsRNA_) when purifying the dsRNA fraction (*p* ≤ 0.004, Supplementary Table S7; Table 1; Figure 3B).

Besides the pre-treatments, the dsRNA construct also had a significant impact on the total RNA extraction efficiency (*p* < 0.0001), contributing to 17% of total variation (Figure 1; Supplementary Table S5). As the ANOVA analysis indicates that this variation is largely independent from the techniques assayed in this study (only 1.6% of variation was assigned to an interaction between the pre-treatments and the dsRNA constructs), the presented experimental data do not allow to conclusively identify the causal factors. We hypothesize that a varying concentration and growth phase of the bacterial cells, as well as the unequal size and sequence of the two dsRNA constructs, might have contributed to the observed results. It may therefore be more appropriate to assign this variation to the cell samples, rather than to the dsRNA constructs. While outside the scope of this paper, follow up studies could make use of biological replicates for each construct to clarify this.

### 4.2 Purification Protocols Yield Enriched and Intact dsRNA

The bacterial total RNA was treated to enrich the dsRNA fraction via either selective precipitation or enzymatic digestion. The results presented in this study show that the type of purification method contributed to under 0.12% of the variation seen in the percentage of retained A_260_ units (%A_260 dsRNA_), with no significant difference found (*p* = 0.9945) (Figure 3A; Supplementary Table S7). Thus, the choice of which purification method to apply should mainly rely on other aspects, such as the cost of the protocol, the ease of use, available equipment, downstream application, and qualitative properties of the purified dsRNAs. Selective precipitation was carried out following the protocol described by Diaz-Ruiz and Kaper (1978). This protocol has the advantage of removing the ssRNA fraction as well as possible DNA remnants that were carried over during the phenol/guanidine RNA extraction. Of all protocols tested, the LiCl based fractionation requires the least hands-on time, reagents, and equipment. Furthermore, this protocol is easily scalable and applicable to a wide range of dsRNA concentrations, while maintaining equal or better dsRNA integrity than the enzymatic digestion alternatives (Figure 2C). Enzymatic removal was carried out using RNase A. The bovine RNase A selectively cleaves ssRNA under high salt conditions, but can degrade dsRNA if the ionic strength becomes too low (Libonati and Sorrentino, 2001). Total RNA was treated with bovine RNase A in a NaCl based digestion buffer with an ionic strength of I = 0.36. This buffer efficiently shielded the dsRNA from degradation, maintaining the intensity of the dsRNA band comparable to that after pre-treatment (Figure 4). While this method does not make use of DNases, the purified dsRNA fraction is expected to contain little or no DNA since the RNA-specific phenol/guanidine extraction was applied twice. In parallel, a combined digestion of ssRNA and dsDNA was attempted as described by Ahn et al. (2019), using bovine RNase A and TURBO™ DNase I. The TURBO™ DNase I was engineered to tolerate higher salt concentrations, maintaining at least 50% of its activity in solutions with up to 200 mM monovalent salt (product manual). The experimental results presented in this paper suggest that the 1x TURBO buffer was not capable of sufficiently shielding the dsRNA from the enzymatic activity of RNase A, with a clear reduction of dsRNA band intensity and increased smearing that were visible for all samples (Figure 4; Figure 2C). Still, the recovered fraction of nucleic acids after treatment was equal to that of other purification methods. Thus, while the dsRNA was qualitatively more degraded, it was quantitatively maintained. The 1x TURBO DNase buffer contains 75 mM monovalent salt (Decker et al., 2019), and increasing the ionic strength of this buffer with additional monovalent salts such as NaCl might further increase the protection of the dsRNA against degradation and thus improve its yield after the double-digestion step.

### 4.3 Pre-Treatments and Biological Samples Affect Composition of Released RNA

In Section 4.1 we determined that the pre-treatment had a major effect on the amount of RNA that could be extracted from the bacterial cells, as it increased RNA yield up to 2.9-fold compared to the control protocol and accounted for 80% of all variation (Supplementary Table S5). We could however not yet make statements regarding the amount of extracted dsRNA. The data on the percentage of A_260_ units retained following purification of dsRNA (%A_260 dsRNA_ **,** Table 1) confirm the previous observation that the pre-treatments differentially promoted the release of total RNA and dsRNA from the cells, as observed by varying band intensities on total RNA gels (Figure 2A). Indeed, while for the no pre-treatment control ∼19% of A_260_ was dsRNA derived, this value significantly decreased (Supplementary Table S7) upon further degradation of the cell wall, to ∼16% for the heating treatment and ∼14% for the lysozyme digestion treatment (Figure 3B). We hypothesize that increasing the porosity of the cell wall may ease the release of large RNAs more than that of smaller molecules such as the dsRNA constructs. An inverse relation between RNA size and release from the cell could also explain why for sonication, where dsRNA fragmentation was observed, the %A_260 dsRNA_ was quasi equal to that of the control treatment (∼19%). Still, these statements are speculative and further research is required to pinpoint the causal factors. Interestingly, the dsRNA constructs (or as explained in Section 4.1, the cell samples) also had a major effect on the %A_260 dsRNA_ (32% of total variation, *p* < 0.0001). As seen in Figures 1, 3C, the *dsDcr2* expressing cells released more total RNA with a lower %A_260 dsRNA_ than the *dsGFP* expressing cells. This could be explained by a higher porosity of the *dsDcr2* expressing cells, along with an inverse relation between RNA size and cellular release. As concluded in Section 4.1, the characteristics of the biological samples and their impact on dsRNA production are outside the scope of this paper but could form an interesting topic for further research.

### 4.4 dsRNA Production Yield is Mainly Determined by the Pre-Treatment Applied

Using the total RNA absorbance and %A_260 dsRNA_ data (Supplementary Table S4; Table 1, respectively), we can calculate the yield of dsRNA per ml of bacterial culture (Table 2; Figure 5). An ANOVA analysis reveals that the pre-treatment was the major determinant of dsRNA yield (*p* < 0.0001), accounting for 94% of observed variation and increasing dsRNA yield up to 3.0-fold compared to the no pre-treatment control (Supplementary Table S8). From these data, we can conclude that researchers should chose the pre-treatment based on their production priorities. If dsRNA band integrity is desired, the heating method will increase the production yield 1.9-fold without compromising the dsRNA qualitatively. On the other hand, if the main goal is the highest yield, the sonication treatment will increase output 3.0-fold. The dsRNA construct (or cell sample) was also found to contribute to 5% of observed dsRNA yield variation. This value is much lower than the 17% seen for the total RNA yield variation, and 32% seen for the %A_260 dsRNA_ variation, suggesting that the purification step reduced the variation derived from the pre-treatments.

### 4.5 dsRNA Production Protocols Are Cost-Effective and Scalable

The experimental data presented in this paper were obtained by treating aliquots of 10 ml bacterial cell culture. To prove the scalability of selected protocols, purified *dsGFP* was produced from a 400 ml bacterial culture. The cells were pre-treated with the heating step and the dsRNA purified via LiCl precipitation. This resulted in intact dsRNA (Figure 2D) with an efficiency of 4.18 µg dsRNA per ml cell culture and a total of 1.67 mg dsRNA. These values are in the range of efficiencies obtained for smaller-scale extractions (5.56 μg/ml). The material cost of production has been calculated at 72.2€ (Supplementary Table S10), for a price of 43.2 €/mg. The prices listed are those available to the authors at the time of writing. They may be subject to change over time or vary between countries. As discussed in the introduction, at the time of writing, researchers in small to medium sized labs have very limited options to obtain custom dsRNA. Ordering from external companies is currently priced at ≥$500 USD for 10 mg. While this price is only slightly higher than that of the presented protocols, it does not account for shipping costs, VAT, nor the average 2–3 weeks waiting period. The value that researchers give to a speedy and *in situ* production of their custom dsRNAs is underlined by the still mainstream usage of small-scale production kits, such as the MEGAscript™ RNAi Kit (Thermo Scientific), with high production costs of ∼$3000 USD for 10 mg. As such, the protocols presented in this paper offer researchers a cost-effective and readily available alternative to produce dsRNAs.

The largest cost of the methods described in this paper stems from the phenol/guanidine-based lysis reagent (40 €/mg of dsRNA) (Supplementary Table S10). Future optimization steps should therefore seek to reduce the required lysis reagent volume or find alternative RNA release methods. Posiri et al. (2013) published a promising “cheap” one-step method that allows the efficient release of total nucleic acids from bacterial cells. Papić et al. (2018) demonstrated the useability of this protocol for large-scale extractions on fed-batch cultures. The major downside of this novel protocol is the co-extraction of large quantities of DNA from the bacterial cells. This requires further purification steps that risk driving up the hands-on time and production cost. The authors did not attempt to isolate the dsRNA fraction limiting the useability of the product for research purposes.

### 4.6 Bacteria-Derived dsRNA is Not Cytotoxic to a *Helicoverpa* Derived Cell Line and Applicable for Cellular Knockdown Experiments

*dsGFP* produced through the protocols presented in this paper was assayed on its cytotoxicity in a lepidopteran derived cell line. *Helicoverpa zea* RP-*Hz*GUT-AW1 cells (Goodman et al., 2004) were transfected with 2.5 μg/ml of either *in vitro* or bacteria-derived dsRNA and their viability and adhesion were assayed. The data indicate that neither cell adhesion or growth (*p* = 0.2970, Figure 6), nor viability (*p* = 0.1359) were significantly affected by the bacterial dsRNA relative to a non-template control. The ability of bacteria-derived dsRNA to induce knockdowns was assessed by transfecting the RP-*Hz*GUT-AW1 cells with two concentrations (0.5 μg/ml or 2.5 μg/ml) of either *dsGFP* or *dsDcr2* targeting the *Helicoverpa Dicer2* gene. *In vitro* synthetized dsRNA was employed as a positive control. The data show that bacteria-derived dsRNA performs on-par with *in vitro* alternatives (Figure 7), for both concentrations tested. The average decrease (±SD) in relative *Dicer2* transcript levels was 39.9 ± 2.5% at 2.5 μg/ml and 34.5 ± 5.4% at 0.5 μg/ml.

The results presented in this paper demonstrate the feasibility, cost-effectiveness, and efficiency of producing bacteria-derived dsRNA in the mg range under laboratory settings. It aims to boost confidence in the useability of bacteria-derived dsRNA for *in vitro* research by demonstrating that it performs as specifically and efficiently as popular synthetic alternatives. Further research could be conducted to assess the useability of bacteria-derived dsRNA for inducing *in vitro* or *in vivo* knockdowns targeting refractory genes, like the Argonaute proteins. In particular, it is possible that dsRNA extracted through the sonication treatment might display distinct knockdown efficiencies due to a broader spectrum of dsRNA sizes, possibly aiding the uptake into the cytoplasm or escape from endocytic bodies.

## Data Availability

The original contributions presented in the study are included in the article/Supplementary Material, further inquiries can be directed to the corresponding author.
