# Supplementary material for: Methods for the Cost-Effective Production of Bacteria-Derived Double-Stranded RNA for in vitro Knockdown Studies
Source: Front Physiol. 2022 Apr 13;13:836106. doi: 10.3389/fphys.2022.836106 (PMC9043282; doi:10.3389/fphys.2022.836106)
Supplement: Supplementary file 1 [file DataSheet1.PDF]

***Supplementary Table 1* – GenBank accession numbers of protein GenBank sequences used for the construction of a phylogenetic maximum likelihood tree of Dicer1 and Dicer2.**

**Species name:**	**Dicer 1**	**Dicer 2**
Helicoverpa armigera	**GFWI01294779**	**GBXD01037200**
Aedes aegypti	AAW48724.1	AAW48725.1
Aedes albopictus	XP 029717928.1	AEX31250.1
Agrilus planipennis	AJF15702.1	AJF15703.1
Apis mellifera	NP 001116485.2	XR 120636.1
Bemisia tabaci	AHY18681.1	AIC07485.1
Blattella germanica	CAX68236.1	CCF23094.1
Bombyx mori	XP 037869731.1	NP 001180543.1
Danaus plexippus plexippus	XP 032528124.1	OWR42902.1
Diabrotica virgifera virgifera	AUM60045.1	AUM60046.1
Drosophila melanogaster	NP 524453.1	NP 001286540.1
Locusta migratoria	BAW35364.1	BAW35365.1
Papilio xuthus	KPJ05873.1	KPJ04293.1
Schistocerca gregaria	BAX36477.1	QVD39336.1
Spodoptera frugiperda	AVK59441.1	AVK59442.1
Spodoptera litura	AHC98016.1	AHC98017.1
Tribolium castaneum	EFA11550.2	NP 001107840.1
Trichoplusia ni	XP 026747931.1	XP 026733953.1
**Outgroup:**	**Dicer:**	
Schizosaccharomyces pombe	NP 588215.2	

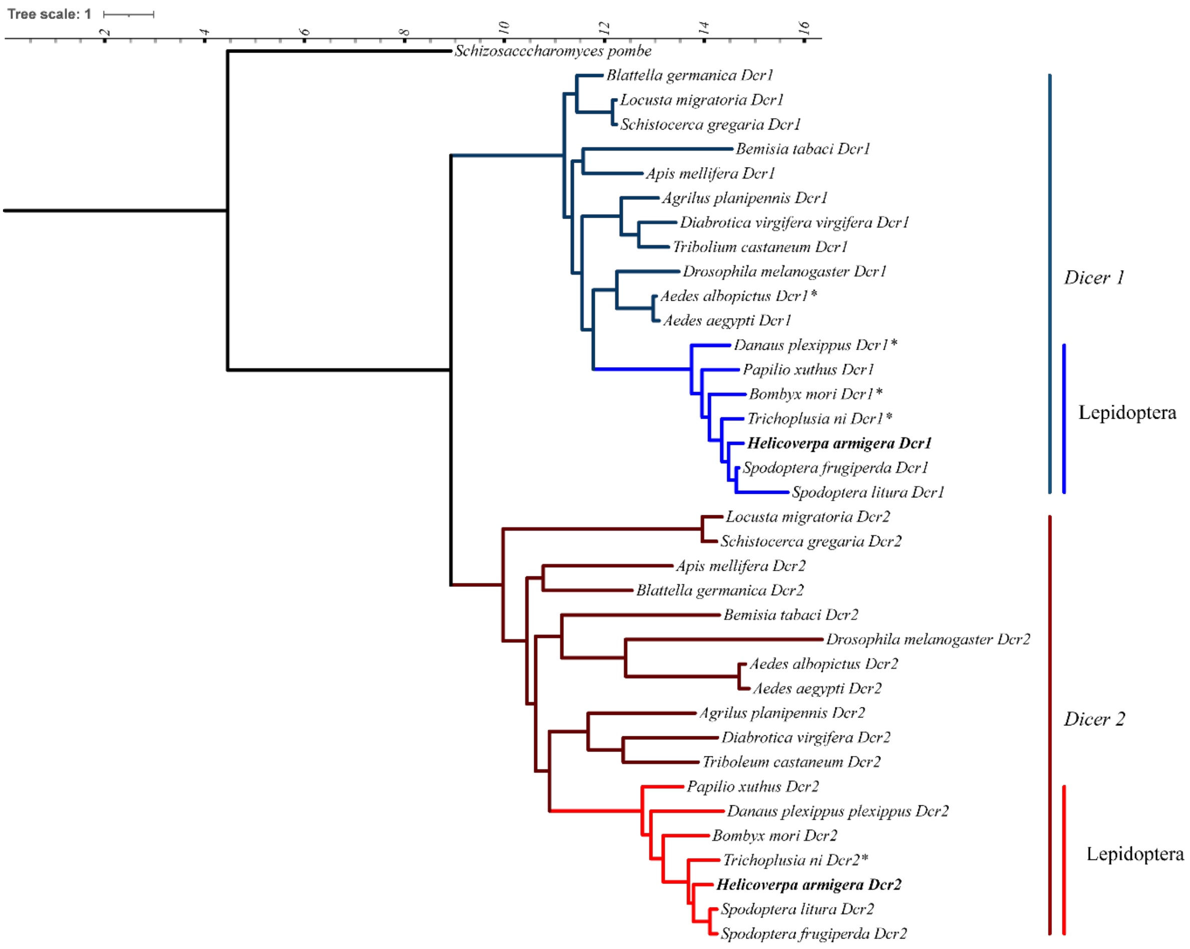

Supplementary Figure 1 **– Maximum likelihood phylogenetic tree of selected insect *Dicer* genes. The identity of discovered *Helicoverpa Dicer* genes was verified by comparing their phylogenetic relationship with known or predicted insect dicers. A dicer protein from the fungus *Schizosaccharomyces pombe* was used as the outgroup. Asterisks indicate NCBI computationally predicted sequences. *Helicoverpa* genes shown in bold.**

Supplementary Table 2 **– List of oligonucleotide primers used during PCR reactions. "qPCR" list shows the primers used for quantitative PCR analysis; "T7 PCR" list shows the primers with T7 overhangs used for RedTaq reactions amplifying the construct used as substrate for in-vitro dsRNA synthesis. "Gibson PCR" list shows the primers with overhangs used for Q5 polymerase reactions amplifying the construct to be inserted into the L4440 vector through Gibson assembly. "Colony PCR" table are the primers used in RedTaq reactions to verify the bacterial strain and plasmids.**

**qPCR**	**Forward**	**Reverse**
Dicer2	GTCGTTGATCACCCAATGCG	TTTTCCTTCGGGCAGACCTC
AK	GGCTCCACCCTCTTGGATTG	GCGTAGATTCCGACACCAGA
ACT	CCAACAGGTAGGTCCTCGTTC	ACAAAGGGCACGGTCAGACT

**T7 PCR**	**Forward**	**Reverse**
GFP	TAATACGACTCACTATAGGGAG AAGGTGATGCTACATACGGAA	TAATACGACTCACTATAGGGAGAT CCCAGCAGCAGTTACAAAC
Dicer2	TAATACGACTCACTATAGGGAG AACGCATTCAGTTGACGCAC	TAATACGACTCACTATAGGGAGAA CTCCTCGCTCGACCTTT

**Gibson PCR**	**Forward**	**Reverse**
Dicer2	CGAGGTCGACGGTATCGATAA ACGCATTCAGTTGACGC	GCTGCAGGAATTCGATATCAGAAC TCCTCGCTCGACCTTTAC

**Colony PCR**	**Forward**	**Reverse**
HT115	TGAAAGCTGGCTACAGGAAGG	ACGCGCTTGTTGAGTTGTTC
GFP		ATCCCAGCAGCAGTTACAAAC
Dicer2		GAACTCCTCGCTCGACCTTT
M13	GTAAAACGACGGCCAGT	

***Supplementary Table 3* – Sequences of the *Helicoverpa armigera Dicer* gene and the green fluorescent protein gene used in this paper. The qPCR amplicon is underlined; the vector insert is indicated in bold.**

Open reading frame of *Helicoverpa armigera Dicer2* from GenBank sequence GBXD01037200
ATGGAGGGCGAGGAATCAGATGCAGTGGAGCACTTCAAGCCGAGGCCTTATCA<br>GGCGCAGCTGGAAGAGATAGCCGTAGAAAACAACACAATCATATATTTGCCAA<br>CAGGTTCTGGCAAGACATTTATCGCGATTTGCCTCATTAAAAGATTTAGGCACG<br>CACTCAAAAAGCCTTGGGGACAGGGAGGCAAAAGAACCTTTTTCTTGGTCAAC<br>ACTGTGCCATTAGTCACACAACAGAAAAAGGTAATAGAAGAGCTGTGTGCAGT<br>TGAAGGCGTTGGTGCTTATAGCGGTGAAAGTGGTGTTGATTATTGGGACAAAG<br>ATAAATGGGATGCCGAACTTGCACAACATCAGGTTATCGTAATGACGAGCCAA<br>ATTTTAAGCGATATGTTGATGCACCAATACATCAGGATCGAAGATATCAACCTT<br>TTGATATTTGACGAATGTCACCACGCCGTCGTTGATCACCCAATGCGTCTTGTA<br>ATGAAACATTTCGAGGTCTGCCCGAAGGAAAATCAGCCGCGAGTTCTTGGTTT<br>AACAGCGACCCTGCTTAACGCAAATGTGAAAACCCATAAGGTCGAGGACACAC<br>TACATGAGTTGGAGATAACGTTCCACGCTAAAATTGCTACCGTTGATGAGTTA<br>GGCAAAGTGCTCAACTATTCAACAAATCCCAACGAAATGGTACAAACGTACAG<br>CAGAACACAGCCGACTGAGGTGTCAGCGCTTGTCATTAGAAAATTAATGGTGC<br>TTCATGGACTAATAGCTAAAATAGAACTACCTTCGCCTCACTCGAAGCACAAT<br>ATTCAGTTAAAGAATTCGCAAAAGAATATCACTGGCGATCCACAGAAGGCAGT<br>GAAAGCCGTCAAAAATATGATTAGTTCCATGATTATGTTCATTGAGGACTTCGG<br>CTTATATGGGGGTGCGTTAGCTATACTTGCTTATATCATCATATTTGAACGATT<br>AAAGCGAAAAACTACGACAAAGGAGGAGGAAATGCTTTATAAAGTAGCTAT**A**<br>**ACGCATTCAGTTGACGCACGGGCAATTCTGCTAAAAGCAATGGATGACAA**<br>**GACTGGTTACGAGAAAATTGTGAAATATTCTTCTGAAAAAGTACTGCTCAC**<br>**TTTAAATGTACTTAAGGAATACAGTCCAAAAGTATTGGAAACTCCTGGTGT**<br>**TACACTGAAAGTAAATAAGTCAAGAAAACCGCTTTCGGCAATTATATTCAC**<br>**ACAACAAAGGTTCACAGCGAAAATTTTGTACAATCTTCTGAAGGACGTAAT**<br>**AGATACCAATCCAGCCGAATATGACTTCCTGAAACATGATTTTATTGTTGG**<br>**TTTTAACGTGAACCCTTACAATAATACTAGAGAGGAATACTATTTGAAAAA**<br>**AGCTAGTCAACAAGCTTTATTGAAGTTTCGGAACAGTGACCTCAACTGCTT**<br>**GATAGCAACAAGCGTAATAGAAGAAGGCATTGATATTCCACAATGTATATT**<br>**GGTCCTGCGTTATGATCCTCCGTTAGAATATCGATCATACATCCAGAGTAA**<br>**AGGTCGAGCGAGGAGTTC**GGAATCGAGTTACGTGATTCTAATAGAGAAGTCA<br>CAACAAAATAATTTCATGAAAAGCTATGAACAGTTCCAGCACACAGAACAAAT<br>TATACAGAGGATATTGATCGGTAGTAGCGACGACCGCAACGAGCCGACATCTA<br>GCGCCATTGATCAGCAACTCTATAAAGATGAAGATGTTGAACCATTCATAACA<br>CCAAATGGAAGTCGGTTATCTTCAGTGTCAGCCATATCTTTGCTAAACCGATAT<br>TGTTCGGTGTTGCCACACGATCAATTCACCGTTATAATCCCAATTTGGATCCAA<br>GAAAGGGTTTTTGATAATAACGGGGTCGAACGCAGGCTGGTCACAATCGTGAT<br>GCCGATTGCATGTCCTATAAAGGAAGAAATAAAGGGCTTGCCAAGGTACAATT<br>TAAAGGCTGCAAAACGGTCGGCGGCGCTAAACGCTTGTATAAAACTGTACGAG<br>GCCGGAGAGTTGGATCCACTAACATTATTGCCCGTTCGTTATACTGCCGTAGAC<br>TTCGACGATACGGATGTCAAATCATGCTTCGCCAACTGGCGCGATGATGACGT<br>GGGTGATGCAGACAGTAATCTACCTTATCCAGGCACCAAGGCACGAGTTCGGA<br>AGCACAAGATTCAGTTCCCACGACTGCTGAACAGTGTTCCAGAAGACGTTTAC

TATTTACACATAATTAAATCCACGACTGCCTTTCAAGAGCCTAAAGATTCCCGC
GAGAAGGCTTTATATAACCTACTGCAGAGAAAAGAGGGTTATGGCTTGTTGAC
TCAGCAACCTCTGCCGCGATTGTGTGAATTCCCAATGTTTATGACGGTTGGAGA
AGTAGCCACTTCCATAGATGTCAACTACGCTGTTATTAAGCTGAATCAAAAGTT
GTTCGAAATTGTCAAAGGATTTCATTACTTTCTATTCGAGCAAGTACTGGCTAT
AGCCAAAAAGTTTTTGGTCTTTGAAGGCAAAGTGAACTGCATGTATGTTGTTCC
CGTGAAAGATGACAATGGATATGATATAGATTGGAATGTTATGACTACCTATA
CTGAGATAGCGCCAGTCCAACCAACTTCTTATGAGGAAAGGCTCTTCATAAAT
GTGACGCCAGAAAACTATAAGGACTGCGTAGTCACGCCTTGGTATCGAGTTCA
GCCAGATAGGTACATTGTGTCAAATGTTTTGGAATATATGACCCCGCAATCTCA
ATTCGATTCAGATTCCTATTTGACTTTCGCCGATTACTATGCCGATAAATATAA
ACTGGAAGTCATAGGAGAAAAAACCCAACCTTTATTAGAGGTGCGTAACATAA
GCTCTAGAATGAACTGCTTATTACCGAGAGCGGCTACTATAAACGCTTTGACA
GACAAGCAAAGGAAACTGGTGTCTGCCTCACAAGGCGACGACAAGACAAGTA
GGGGCTTTGCCGAGGTATTTGTCGCTGAATTTTGTGTCAAATACGATTATCCTG
GCGTTCTGTGGTACAAGGCTATAATGTTGCCTAGTATTATACACAGAGTATTTA
TGTTGTTGGTAGCGCACGAACTGTTAACTGAAATAGCTGAAAAGACAAAATTC
GGAACTCCTATTCGCAAAAAATCTACCGACTGGCTTCCAGTAAAGGGAAGTAT
ACTAATCGCAACAAACTCACTGCTTGCACAAGTTGAAGAGCCAACACCTATAA
ATTCAGTAGACAGGATTAACAATGCTGTGGACGATGACAGTAGTAGACGTCCT
AATGTACTTTCTATAAAAGACAGTTTGTACCGACTGCAACAAAAGAAGCTCAG
TAAGGAATATCCATGGGATGAAAGAATGGAACCGATCGACATAGAACGTAAC
TTGTCGTCTGTGTCGGTAATGGACGTGGAATGTTATGACGAGTTCGTGTCGGCG
CCTCTAATCGCATCCCCACGTTCCAACTACAATGTGAGATCACCGCAGTCTGTT
TATACACATTCTGCGGCTATATCTGCGCCTCCTGCTAAGTACAACGACCAAATA
AAGCTTCTCAAAGCATCAGCCACCGGCAAAGGACCAGAATTACGGGATATTCT
AGCTGCGTTAACAACCATAAAATCTCACGATACCTTCAATTTGGAGCGAGTTG
AGACACTCGGAGATTCATTCCTTAAGTTCGCAGCCAGCTTATATCTGTACCATA
AGTTCCCGAAGCTCAATGAAGGCCAACTAACTAATATTAAATGTCGATTAATT
AGTAACAGAAACCTTTACTATGCTGGCGAGCGATTCAATCTGGGAGGCCGAAT
GAAAATAGACCAGTTCAGTCCAAGGAATGATTTCATGCTGCCAGGGTTCTTTG
CGCCAAAGGAGGTTGAAGATTTTATTGCTGAGAAACAGATTCGTCCGATATTC
CTGATCGGAGTACAATTCCCGCATTCTGAGGTCCTTGATGGCAATCTATCGAAG
GAAAGCATGGATATGGTTCGGGATAGATATACTGACTGCGACGGTGCCGCGGA
GATCGAACCCCGTGATCGCGCTCAAAACGCAATGCAGCTGTATGTTCATTCCC
AGGCCGTGCCCGATAAGTCAGTGGCTGATTGCGTTGAAGCACTCATCGGTACT
TACTTGTTGAGCGGTGGACTGATAGGGGCCATCAAAGTCATCGAGTGGATGAG
GATCATACCCCCACAGGATGATTTTGCGGTGTATTTACACTTGCGTGTACCGAC
CGCCATTACAGATAAGAAAGCGACCGAGCAAGACATCGATTTCCTATTGAGAC
ATTGCAGAGATGATGTCGAGAAAATCTTAAATTACAAGTTTAATGACCCGTCTT
TCCTATTGGAGGCGTTATCCCATCCATCATATATTCGGAACCGACTGACTCGTT
CATACGAACGAATGGAGTTCCTAGGCGACGCCATTTTGGATTTCCTAATAACAT
CACACATTTTTGAACATTGCGGGGAATTGAAGCCTGGTGAAATGACCGACTTA
AGATCAGCGCTAGTGAATAATGTCACTTTTGCCTCGTATGTGGTCAAACTTGGA
CTACACAAATTCCTTTGCTCTGAGCTAAATCCGACTTTAGACACGGCTATCATG
ACGTTCGTCGAACATCAAATACAGAGGGACCACGAGATTGTTGAAGATGTACT
CTATATGATGGACGAGGAAGAATGTAGTCTTGCGGAATATATTGAAGTTCCTA
AGGTGCTGAGTGACATATTCGAAGCGCTAGTGGGCGCGATATATTTGGACTGC
GGCGGCGACCTACAAGTTGTATGGTCGGTAGTTTACCGTATAATGTGTAAGGA
AATACATGACTTCTCGTGCCGTATACCGCAACAGCCCGTTAAGATATTGTACGA

GAAAATACACGCGTGTCCCACTTTCAGGAAACCAGAAGTCATCGATCCTGACA<br>TTCCAAAGATACGGATAGGGGTTACAATCACGAAGAATGACTGGCAACACACA<br>GTCTATGGCATTGGTCGAAACAAGGCTCAAGCAAAACGAGCCGCCGCTAAAAT<br>GGCTCTTAAGGTCTTGGGCCTTTAA
Insert sequence of the L4440-*GFP* vector construct
AAGGTGATGCTACATACGGAAAGCTTACCCTTAAATTTATTTGCACTACTGGAA<br>AACTACCTGTTCCATGGCCAACACTTGTCACTACTTTCTCTTATGGTGTTCAATG<br>CTTTTCCCGTTATCCGGATCATATGAAACGGCATGACTTTTTCAAGAGTGCCAT<br>GCCCGAAGGTTATGTACAGGAACGCACTATATCTTTCAAAGATGACGGGAACT<br>ACAAGACGCGTGCTGAAGTCAAGTTTGAAGGTGATACCCTTGTTAATCGTATC<br>GAGTTAAAAGGTATTGATTTTAAAGAAGATGGAAACATTCTCGGACACAAACT<br>CGAGTACAACTATAACTCACACAATGTATACATCACGGCAGACAAACAAAAGA<br>ATGGAATCAAAGCTAACTTCAAAATTCGTCACAACATTGAAGATGGATCCGTT<br>CAACTAGCAGACCATTATCAACAAAATACTCCAATTGGCGATGGCCCTGTCCTT<br>TTACCAGACAACCATTACCTGTCGACACAATCTGCCCTTTCGAAAGATCCCAAC<br>GAAAAGCGTGACCACATGGTCCTTCTTGAGTTTGTAACTGCTGCTGGGAT

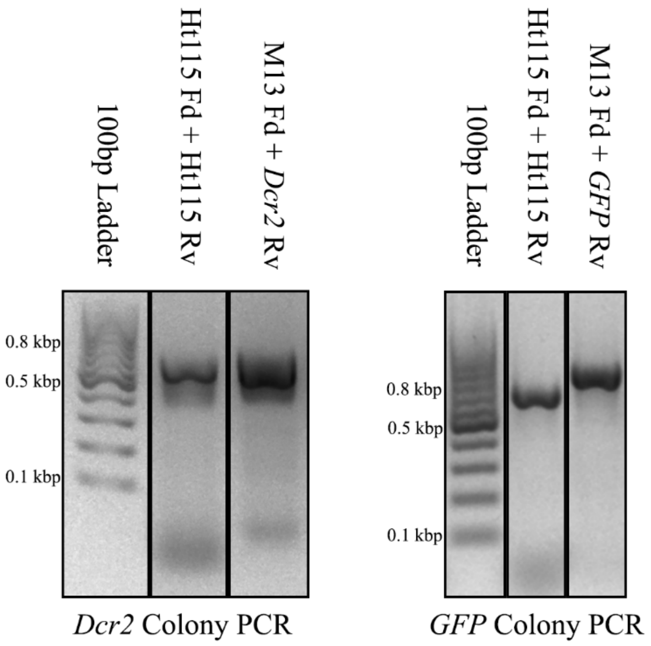

Supplementary Figure 2 **– Agarose gel electrophoresis of RedTaq PCR products for the identification of bacterial colonies. HT115 bacteria transformed with the L4440-*GFP* or L4440-*HaDicer2* plasmids were plated onto selective LB agar plates. Individual colonies from HT115 + *L4440-HaDicer2* (left) or + *L4440-GFP* (right) were picked and used as substrates for RedTaq PCR reactions as described in the materials and methods section. The RedTaq PCR products were run on a 1% agarose gel for 40 minutes. Expected band sizes: HT115 Fd + Rv 609 bp; M13 Fd + *GFP* Rv 727 bp. M13 Fd + *Dicer2* Rv 618 bp.**

Supplementary Table 4 **– Absorbance at 260nm values for total RNA samples.**

A_{260}		***GFP***				***Dicer2***			
		**1**	**2**	**3**	**4**	**1**	**2**	**3**	**4**
**Pre-treatment**	**Lysozyme**	25.77	25.05	27.42	26.27	43.10	41.45	39.41	35.57
	**Sonication**	49.25	47.20	46.01	49.65	57.40	61.48	63.56	58.80
	**Heating**	31.10	32.32	36.96	32.98	48.11	49.27	49.25	50.74
	**Control**	18.83	14.52	15.01	14.94	21.56	22.34	21.66	21.98

Supplementary Table 5 **– Results of a two-way ANOVA with Tukey's multiple comparisons test on the total RNA extraction yield (data from *Supplementary Table 4*), for two dsRNA constructs.**

Source of Variation	% of total variation	*p* value	*p* value summary
**Interaction**	1.630	0.0004	***
**Pre-treatment**	79.79	<0.0001	****
**dsRNA construct**	17.08	<0.0001	****

Tukey's multiple comparisons test	Mean diff.	95.00% CI of diff.	Adjusted *p* value	Summary
**Control vs. Lysozyme**	-10.12	-12.14 to -8.097	<0.0001	****
**Control vs. Heating**	-16.23	-18.26 to -14.21	<0.0001	****
**Control vs. Sonication**	-25.50	-27.53 to -23.48	<0.0001	****
**Lysozyme vs. Heating**	-6.110	-8.134 to -4.086	<0.0001	****
**Lysozyme vs. Sonication**	-15.38	-17.41 to -13.36	<0.0001	****
**Heating vs. Sonication**	-9.271	-11.30 to -7.247	<0.0001	****

Supplementary Table 6 **– Absorbance at 260nm values for purified dsRNA samples.**

A_{260}		*GFP*			*Dicer2*		
		**Purification method**					
		**LiCl precipitation**	**NaCl buffer**	**TURBO buffer**	**LiCl precipitation**	**NaCl buffer**	**TURBO buffer**
**Pre-treatment**	**Lysozyme**	3.164	3.812	3.775	2.567	3.052	2.910
	**Sonication**	4.716	4.528	4.543	3.944	4.016	4.139
	**Heating**	4.085	4.051	3.684	3.463	3.130	3.635
	**Control**	4.868	4.704	4.632	4.048	3.777	3.899

Supplementary Table 7 **– Results of a repeated measure two-way ANOVA with Tukey's multiple comparisons test on the percentage of retained A260 units after purification (data from *Table 1*), for two dsRNA constructs.**

**Source of Variation**	**% of total variation**	***p* value**	***p* value summary**
**Interaction**	4.951	0.0997	ns
**Pre-treatment**	60.15	0.0007	***
**Purification method**	0.1178	0.9945	ns
**dsRNA construct**	31.87	<0.0001	****

**Tukey's multiple comparisons test**	**Mean diff.**	**95.00% CI of diff.**	**Adjusted *p* Value**	**Summary**
**Lysozyme vs. Heating**	-2.083	-5.034 to 0.8677	0.1555	ns
**Lysozyme vs. Sonication**	-4.983	-7.039 to -2.928	0.0011	**
**Lysozyme vs. Control**	-4.933	-7.496 to -2.370	0.0033	**
**Heating vs. Sonication**	-2.900	-4.120 to -1.680	0.0012	**
**Heating vs. Control**	-2.850	-4.394 to -1.306	0.0040	**
**Sonication vs. Control**	0.05000	-1.191 to 1.291	0.9987	ns

Supplementary Table 8 **– Results of a repeated measure two-way ANOVA with Tukey's multiple comparisons test on dsRNA production yield (data from *Table 2*), for two dsRNA constructs.**

Source of Variation	% of total variation	*p* value	*p* value summary
**Interaction**	0.7593	0.2633	ns
**Pre-treatment**	93.56	<0.0001	****
**Purification method**	0.02144	0.9935	ns
**dsRNA construct**	4.927	0.0002	***

Tukey's multiple comparisons test	Mean diff.	95.00% CI of diff.	Adjusted *p* value	Summary
**Control vs. Lysozyme**	-0.9550	-1.572 to -0.3385	0.0086	**
**Control vs. Heating**	-2.688	-3.503 to -1.874	0.0003	***
**Control vs. Sonication**	-5.762	-6.259 to -5.265	<0.0001	****
**Lysozyme vs. Heating**	-1.733	-2.836 to -0.6306	0.0081	**
**Lysozyme vs. Sonication**	-4.807	-5.382 to -4.231	<0.0001	****
**Heating vs. Sonication**	-3.073	-3.777 to -2.369	<0.0001	****

Supplementary Table 9 **– Collection of multiple *t*-test analyses on the *Dicer2* ddCt values for all knockdown conditions tested.**

Condition		Mean of *dsGFP*	Mean of *dsDcr2*	Difference ± SE	T ratio	*p* value	Summary
**0.5 µg/ml**	**MEGA**	1.003	0.5828	0.4200 ± 0.06049	6.944	0.0004	***
	**B/E**	1.003	0.6595	0.3433 ± 0.04816	7.128	0.0004	***
	**B/L**	1.001	0.7233	0.2773 ± 0.03411	8.129	0.0002	***
	**C/E**	1.001	0.6248	0.3765 ± 0.05604	6.719	0.0005	***
	**C/L**	1.001	0.6835	0.3170 ± 0.01852	17.11	<0.0001	****
**2.5 µg/ml**	**MEGA**	1.001	0.5674	0.4336 ± 0.05337	8.124	0.0002	***
	**B/E**	1.005	0.5966	0.4086 ± 0.08362	4.887	0.0027	**
	**B/L**	1.001	0.6043	0.3970 ± 0.03312	11.98	<0.0001	****
	**C/E**	1.002	0.5982	0.4042 ± 0.04039	10.01	<0.0001	****
	**C/L**	1.001	0.6368	0.3645 ± 0.03949	9.230	<0.0001	****

Supplementary Table 10 **– Total cost of consumables for the production and purification of dsRNA from 400 ml bacterial culture, using the heating or sonication pre-treatments and LiCl purification method.**

Component	Amount		Price		Cost (€)
LB broth powder	10	g	34.24	€/kg	0.34
Ampicillin	20	mg	161.98	€/25g	1.30
Tetracycline	5	mg	65.2	€/25g	0.13
IPTG	96	mg	74	€/5g	1.42
15 ml falcon tubes	4	u	87.50	€/1000u	0.35
50 ml falcon tubes	2	u	50	€/500u	0.28
QIAzol	40	ml	308.7	€/200ml	61.74
Chloroform	8	ml	4.98	€/l	0.04
Isopropanol	13.5	ml	13.6	€/2.5l	0.07
Ethanol	21	ml	9.7	€/l	0.20
Liquid Nitrogen	1	l	0.075	€/l	0.08
RNase-free Water	15	ml	67.56	€/l	1.01
LiCl (8M)	10	ml	52.34	€/100 ml	5.23
**TOTAL (€):**					72.20
